# Nitric oxide signaling through three receptors regulates virulence, biofilm formation, and phenotypic heterogeneity of *Legionella pneumophila*

**DOI:** 10.1128/mbio.00710-24

**Published:** 2024-04-29

**Authors:** Sarah Michaelis, Tong Chen, Camille Schmid, Hubert Hilbi

**Affiliations:** 1 Institute of Medical Microbiology, University of Zürich, Zürich, Switzerland; Yale University School of Medicine, New Haven, Connecticut, USA

**Keywords:** amoeba, *Acanthamoeba*, biofilm, cell-cell communication, flagellum, host-pathogen interaction, inter-kingdom signaling, intracellular replication, *Legionella*, macrophage, microcolony, nitric oxide, phenotypic heterogeneity, quorum sensing, timer

## Abstract

The causative agent of Legionnaires’ disease, *Legionella pneumophila*, is an environmental bacterium, that replicates in macrophages, parasitizes amoeba, and forms biofilms. *L. pneumophila* employs the *Legionella* quorum sensing (Lqs) system and the transcription factor LvbR to control various bacterial traits, including virulence and biofilm architecture. LvbR negatively regulates the nitric oxide (NO) receptor Hnox1, linking quorum sensing to NO signaling. Here, we assessed the response of *L. pneumophila* to NO and investigated bacterial receptors underlying this process. Chemical NO donors, such as dipropylenetriamine (DPTA) NONOate and sodium nitroprusside (SNP), delayed and reduced the expression of the promoters for flagellin (P*_flaA_*) and the 6S small regulatory RNA (P*_6SRNA_*). Marker-less *L. pneumophila* mutant strains lacking individual (Hnox1, Hnox2, or NosP) or all three NO receptors (triple knockout, TKO) grew like the parental strain in media. However, in the TKO strain, the reduction of P*_flaA_* expression by DPTA NONOate was less pronounced, suggesting that the NO receptors are implicated in NO signaling. In the Δ*nosP* mutant, the *lvbR* promoter was upregulated, indicating that NosP negatively regulates LvbR. The single and triple NO receptor mutant strains were impaired for growth in phagocytes, and phenotypic heterogeneity of non-growing/growing bacteria in amoebae was regulated by the NO receptors. The single NO receptor and TKO mutant strains showed altered biofilm architecture and lack of response of biofilms to NO. In summary, we provide evidence that *L. pneumophila* regulates virulence, intracellular phenotypic heterogeneity, and biofilm formation through NO and three functionally non-redundant NO receptors, Hnox1, Hnox2, and NosP.

**IMPORTANCE:**

The highly reactive diatomic gas molecule nitric oxide (NO) is produced by eukaryotes and bacteria to promote short-range and transient signaling within and between neighboring cells. Despite its importance as an inter-kingdom and intra-bacterial signaling molecule, the bacterial response and the underlying components of the signaling pathways are poorly characterized. The environmental bacterium *Legionella pneumophila* forms biofilms and replicates in protozoan and mammalian phagocytes. *L. pneumophila* harbors three putative NO receptors, one of which crosstalks with the *Legionella* quorum sensing (Lqs)-LvbR network to regulate various bacterial traits, including virulence and biofilm architecture. In this study, we used pharmacological, genetic, and cell biological approaches to assess the response of *L. pneumophila* to NO and to demonstrate that the putative NO receptors are implicated in NO detection, bacterial replication in phagocytes, intracellular phenotypic heterogeneity, and biofilm formation.

## INTRODUCTION

The causative agent of Legionnaires’ disease, *Legionella pneumophila*, is an environmental bacterium, which colonizes biofilms (1, 2) and replicates intracellularly in protozoa (3–5). Upon inhalation of *Legionella*-laden aerosols, the opportunistic pathogen reaches the lung and replicates in alveolar macrophages, thus triggering fulminant pneumonia (6, 7). Many mechanistic aspects of intracellular replication in protozoa and macrophages are evolutionarily conserved, and *L. pneumophila* is sophisticatedly adapted for its intracellular life (8).

*L. pneumophila* orchestrates intracellular replication and other interactions with eukaryotic host cells through a type IV secretion system (T4SS) termed Icm/Dot (intracellular multiplication/defective organelle trafficking) (9–11). The Icm/Dot T4SS translocates the striking number of more than 300 different “effector proteins”, which manipulate a plethora of cellular processes and promote the formation of a membrane-bound, ER-associated replication compartment, the *Legionella*-containing vacuole (LCV) (12–19).

**Fig 1:**
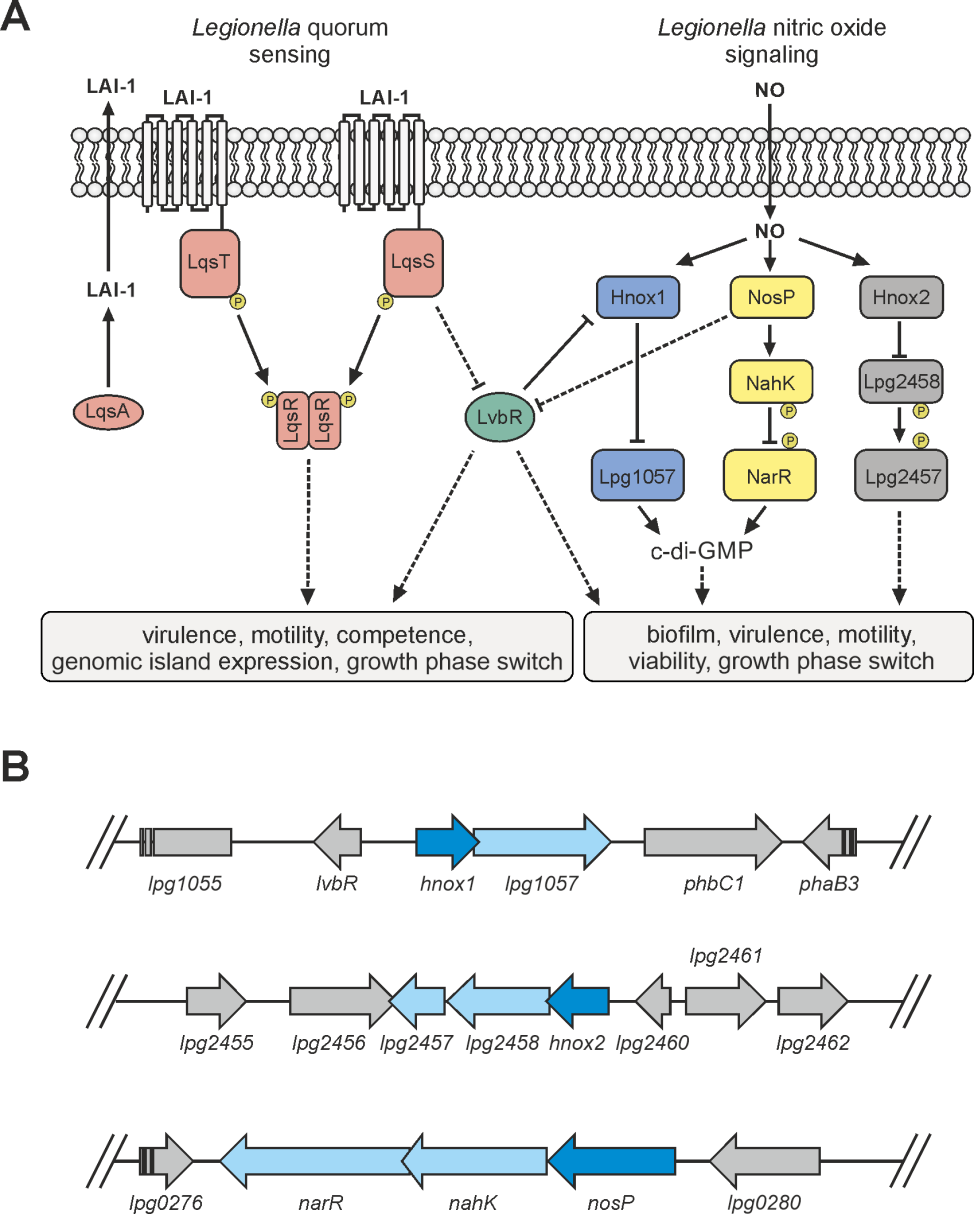
The *L. pneumophila* Lqs-LvbR regulatory network, NO signaling, and genomic organization of NO receptor genes. (**A**) The Lqs (*Legionella* quorum sensing) system produces, detects, and responds to the α-hydroxyketone compound LAI-1 (*Legionella* autoinducer-1) to regulate various traits of *L. pneumophila*. The Lqs system comprises the autoinducer synthase LqsA, the cognate membrane-bound sensor histidine kinases LqsS and LqsT, and the receiver domain-containing response regulator LqsR. The transcription factor LvbR (*Legionella* virulence and biofilm regulator) is regulated by LqsS, regulates the nitric oxide (NO) sensor Hnox1, and, through the diguanylate cyclase Lpg1057, cyclic di-guanosine monophosphate (c-di-GMP) signaling. The NO sensor NosP regulates LvbR and signals through the two-component system (TCS) NahK-NarR to regulate c-di-GMP levels. The NO sensor Hnox2 is upstream of a TCS comprising the histidine kinase Lpg2458 and the CheY-like single domain response regulator Lpg2457, the downstream signal of which is not known. (**B**) Genomic organization of the three NO receptor genes *nosP*, *hnox1*, and *hnox2*: *nosP* (*lpg0279*) forms an operon with *nahK* and *narR*, *hnox1* (*lpg1056*) with *lpg1057* (divergently transcribed from *lvbR*), and *hnox2* (*lpg2459*) with *lpg2458* and *lpg2457*, respectively.

*L. pneumophila* inter-kingdom interactions are not only mediated by bacterial effector proteins but also through the low molecular weight signaling molecule LAI-1 (*Legionella* autoinducer-1, 3-hydroxypentadecane-4-one), which is produced and detected by the Lqs (*Legionella* quorum sensing) system (20–22) (Fig. 1A). LAI-1 and components of the Lqs system modulate the migration of *Dictyostelium discoideum* amoebae, macrophages, or epithelial cells (23) regulate the movement of *Acanthamoeba castellanii* amoebae through *L. pneumophila* biofilms (24) and promote intracellular replication of *L. pneumophila* in macrophages (25). The Lqs system also regulates many traits of *L. pneumophila*, including virulence (22, 26), motility (27), phenotypic heterogeneity of bacterial subpopulations (28–30), natural competence (31), expression of a “genomic island” (32), temperature-dependent cell density (33), and the switch from the replicative, non-virulent to the stationary, virulent and motile form (33, 34).

The signaling molecule LAI-1 is produced by the pyridoxal-5′-phosphate (PLP)-dependent autoinducer synthase LqsA (25, 32, 35), secreted and delivered by bacterial outer membrane vesicles (25), and detected by the two paralogous *L. pneumophila* sensor histidine kinases LqsS (27, 32) and LqsT (27, 31) (Fig. 1A). Upon binding of LAI-1, LqsS and LqsT convergently relay a phosphorylation signal to the prototypic response regulator LqsR (27, 34, 36). LqsR contains a canonical aspartate phosphate receiver domain, dimerizes upon sensor kinase-mediated phosphorylation, and possesses a putative nucleotide-binding output domain (37, 38) (Fig. 1A).

The Lqs system is integrated into and crosstalks with other regulatory systems of *L. pneumophila*, such as sensor histidine kinase-response regulator two-component systems (22, 39) and the cyclic di-guanosine monophosphate (c-di-GMP) signaling network (40) (Fig. 1A). The sensor kinase LqsS negatively regulates the pleiotropic transcription factor LvbR (*Legionella* virulence and biofilm regulator), which itself negatively regulates Hnox1 (haem-nitric oxide/oxygen binding domain) (32, 41), an inhibitor of the GGDEF/EAL domain-containing diguanylate cyclase (DGC) Lpg1057 (42, 43) (Fig. 1A). Accordingly, LqsS likely promotes the production of c-di-GMP by negatively regulating a diguanylate cyclase inhibitor (40, 41).

Another small molecule implicated in bacterial signaling is nitric oxide (NO). NO is a membrane-permeable diatomic gas, which is toxic at high (µM) concentrations and serves as a chemical cue for bacteria as well as for eukaryotic cells at low (nM-µM) concentrations (44, 45). The Lqs system is linked to NO signaling through LqsS, LvbR, and the NO-binding protein Hnox1 (42) (Fig. 1A). Purified Hnox1 and its homolog Hnox2 bind NO, and Hnox1 inhibits the DGC activity of purified Lpg1057 and reduces c-di-GMP production (42). Analogously, purified NosP (NO sensing protein) binds NO, which activates autophosphorylation of the sensor kinase NahK (NosP-associated histidine kinase) and subsequent phospho-transfer to the response regulator NarR (NosP-associated response regulator) (46) (Fig. 1A). NarR is a DGC/PDE (phosphodiesterase) bifunctional enzyme, which synthesizes and degrades c-di-GMP. Upon phosphorylation, the DGC activity is reduced, resulting in lower levels of c-di-GMP and impaired biofilm formation (43, 46–48). While Hnox1 and NosP have been biochemically implicated in NO-binding and c-di-GMP signaling *in vitro*, the response of *L. pneumophila* to NO and the role of *L. pneumophila* NO sensors have been barely characterized *in vivo*.

In this study, we employ pharmacological, genetic, and cell biological approaches to determine the response of *L. pneumophila* to NO and to demonstrate that the NO receptors Hnox1, Hnox2, and NosP are implicated in NO detection by the pathogen, replication in phagocytes, phenotypic heterogeneity, and biofilm formation.

## RESULTS

### Chemical NO generators regulate the *flaA* and *6SRNA* promoters in *L. pneumophila*

The response of *L. pneumophila* to chemically produced NO has not been assessed in detail. To pharmacologically test whether *L. pneumophila* responds to NO, we employed the chemical NO generators sodium nitroprusside (SNP) (Fig. S1A) and dipropylenetriamine (DPTA) NONOate (Fig. S1B). SNP and DPTA NONOate release at a given concentration approximately 1/1,000 (SNP) or 2/1,000 (DPTA NONOate) NO molecules, respectively (i.e., 1 µM SNP = ~1 nM NO) (49). The compounds were used to study the effects of NO on the expression of *gfp* under the control of the promoters for flagellin (P*_flaA_*), the 6S small regulatory RNA (P*_6SRNA_*), the effector protein SidC (P*_sidC_*), or the NO receptor Hnox1 (P*_hnox1_*), respectively.

**Fig 2:**
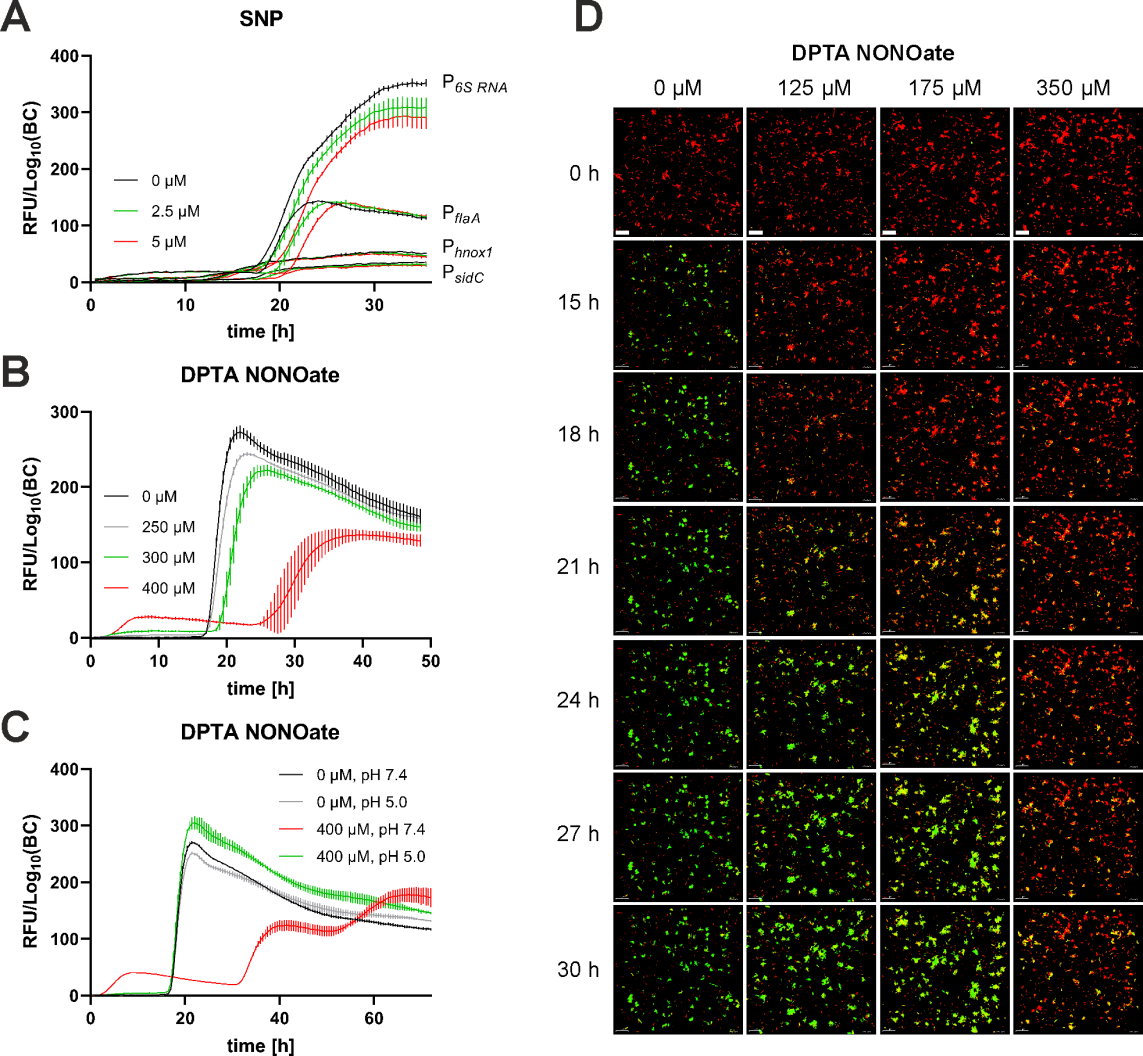
NO delays the expression of P*_flaA_-gfp* and P*_6SRNA_-gfp* in *L. pneumophila. L. pneumophila* JR32 harboring (**A**) P*_flaA_-gfp* (pCM009), P*_6SRNA_-gfp* (pRH049), P*_sidC_-gfp* (pRH035), or P*_hnox1_-gfp* (pRH026) reporter constructs or (**B, C**) P*_flaA_-gfp* (pCM009) was grown in AYE medium for 18 h at 37°C and inoculated in AYE medium at an initial OD_600_ of 0.2. GFP fluorescence and OD_600_ were measured over time using a microplate reader. Promoter activity is inferred by *gfp* expression levels, denoted as relative fluorescence units divided by log_10_(bacterial counts) [RFU/Log_10_(BC)]. Strains were grown at 30°C (**A**) without (black) or with 2.5 µM (green) or 5.0 µM SNP (red), (**B**) without (black) or with 250 µM (gray), 300 µM (green), or 400 µM DPTA NONOate (red), or (**C**) without (black) or with 400 µM DPTA NONOate (red), or without (gray), or with 400 µM spent DPTA NONOate (green). Data shown (**A–C**) are means and standard deviations of technical triplicates and are representative of three independent experiments. (**D**) *L. pneumophila* JR32 expressing P*_tac_-mCherry* and P*_flaA_-gfp* (pSN07) was grown in AYE medium for 21–22 h at 37°C, immobilized in AYE/0.5% agarose in 8-well ibidi dishes and let form microcolonies for 24 h at 30°C. After the addition of DPTA NONOate (0, 125, 175, or 350 µM), P*_tac_-mCherry* and P*_flaA_-gfp* expression were recorded by time-lapse microscopy for 30 h, and microcolony formation was analyzed by 3D reconstruction (Imaris). Scale bars: 20 µm. Data shown are means and standard deviations of technical triplicates and representative of three biological replicates.

SNP was added to *L. pneumophila* JR32 expressing *gfp* under the control of P*_flaA_*, P*_6SRNA_*, P*_sidC_*, or P*_hnox1_*, and the relative fluorescence (RFU) normalized to bacterial counts was assessed in AYE medium (Fig. 2A). Under these conditions, 2.5 µM and 5 µM SNP dose-dependently delayed the production of GFP under the control of P*_flaA_* or P*_6SRNA_* but did not affect the production of GFP under the control of P*_sidC_* or P*_hnox1_*. The SNP-dependent delay in P*_flaA_*-controlled production of GFP was observed in the parental *L. pneumophila* strain JR32, as well as in the Δ*lqsR* mutant strain (Fig. S2A), which shows an overall reduced P*_flaA_* expression (27). Similar results were obtained upon quantifying the production of GFP under the control of P*_flaA_* (Fig. S2B and C) or P*_6SRNA_* (Fig. S2D and E) by flow cytometry. After 20 h of growth, 2.5 µM and 5 µM SNP significantly reduced the production of GFP under the control of P*_flaA_* or P*_6SRNA_*, and after 22 h or 24 h of growth, 5 µM SNP was still effective.

To confirm the results obtained with SNP, we treated *L. pneumophila* JR32 expressing *gfp* under the control of P*_flaA_* with up to 400 µM of the NO-generating chemical DPTA NONOate in AYE medium (Fig. 2B). Under the conditions used, DPTA NONOate dose-dependently delayed the production of GFP under the control of P*_flaA_*, similar to what was observed with SNP. At a concentration of 400 µM, DPTA NONOate not only delayed P*_flaA_*-dependent *gfp* expression but also significantly reduced the production of GFP (Fig. 2B). DPTA NONOate dissolved in PBS pH 5.0 quantitatively releases NO upon incubation at room temperature for 1 h. This “spent” DPTA NONOate no longer affected the production of P*_flaA_*-controlled GFP (Fig. 2C), and therefore, the release of NO underlies the biological activity of the compound on *L. pneumophila*.

In a different approach, we tested the effect of DPTA NONOate on the expression of *gfp* under the control of P*_flaA_* in sessile microcolonies (Fig. 2D). Under these conditions, the treatment with DPTA NONOate also caused a dose-dependent delay in the P*_flaA_*-dependent *gfp* expression in the course of 30 h of microcolony formation. In summary, the NO-generating compounds SNP and DPTA NONOate dose-dependently delay the expression of P*_flaA_* or P*_6SRNA_* in *L. pneumophila* grown in planktonic or sessile form in AYE medium. These results indicate that *L. pneumophila* detects and responds to NO in the nanomolar concentration range.

### Construction of marker-less *L. pneumophila* mutant strains lacking NO receptors

**Fig 3:**
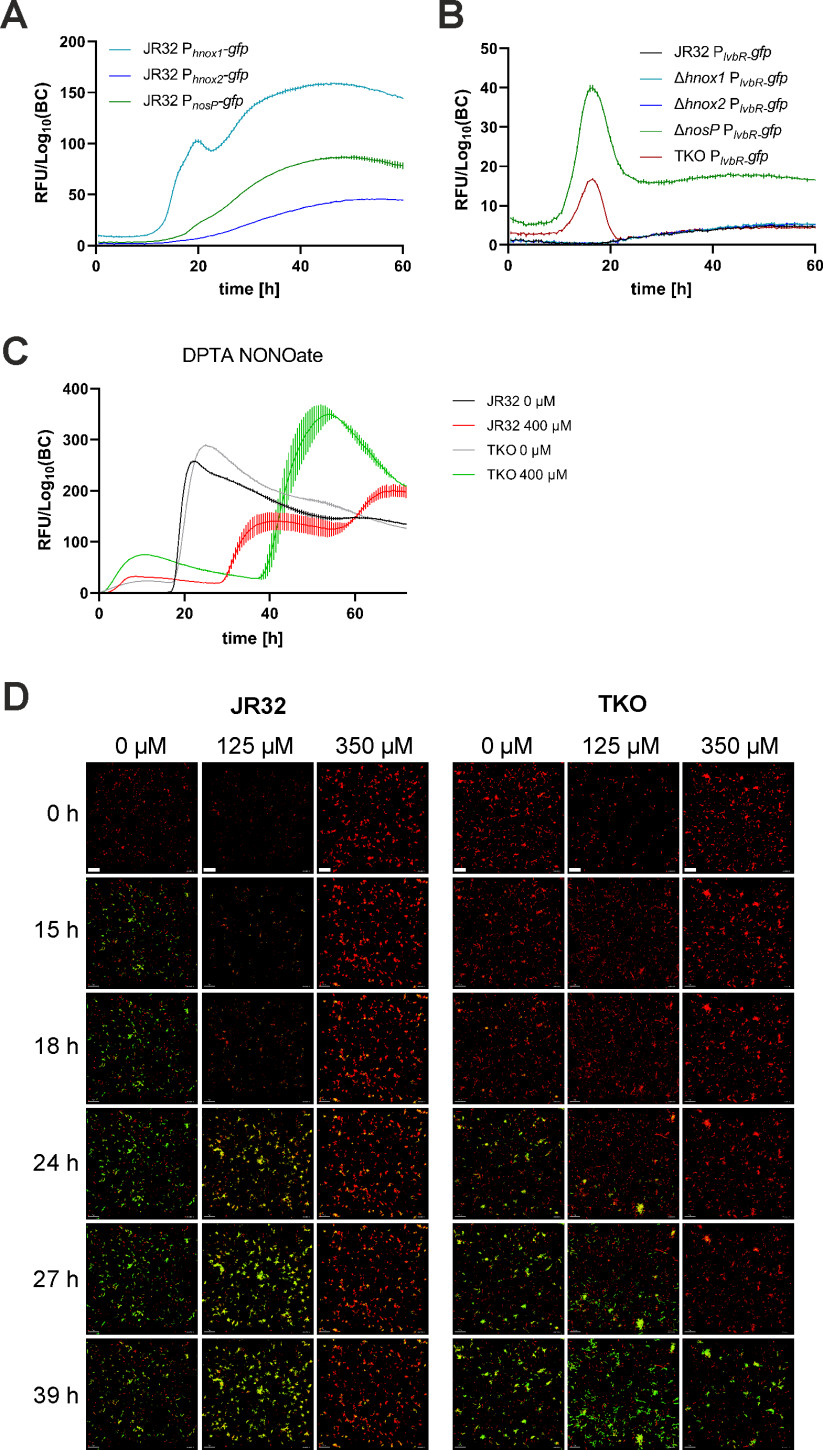
*L. pneumophila* strains lacking NO receptors are less responsive to NO. (**A**) *L. pneumophila* JR32 harboring P*_hnox1_-gfp* (pRH026), P*_hnox2_-gfp* (pCS031), and P*_nosP_-gfp* (pCS032) or (**B**) *L. pneumophila* JR32, ∆*hnox1*, ∆*hnox2*, ∆*nosP*, and TKO mutant strains harboring P*_lvbR_-gfp* (pRH023) were grown in AYE medium for 18 h at 37°C and inoculated in AYE medium at an initial OD_600_ of 0.2. Strains were grown at 30°C, and OD_600_ and GFP fluorescence were measured over time using a microplate reader. Promoter activity is inferred *via gfp* expression levels, denoted as relative fluorescence units divided by log_10_(bacterial counts) [RFU/Log_10_(BC)]. The data shown are means and standard deviations of technical triplicates and are representative of three independent experiments. (**C**) *L. pneumophila* JR32 (black/red) or TKO strains (grey/green) harboring P*_flaA_-gfp* (pCM009) were grown in AYE medium for 18 h at 37°C and inoculated in AYE medium at an initial OD_600_ of 0.2. Strains were grown without (black/gray) or with 400 µM DPTA NONOate (red/green) at 30°C, and OD_600_ and GFP fluorescence were measured over time using a microplate reader. Promoter activity is inferred *via gfp* expression levels, denoted as relative fluorescence units divided by log_10_(bacterial counts) [RFU/Log_10_(BC)]. The data shown are means and standard deviations of technical triplicates and are representative of three independent experiments. (**D**) *L. pneumophila* JR32 or TKO mutants expressing P*_tac_-mCherry* and P*_flaA_-gfp* (pSN07) were grown in AYE medium for 21–22 h at 37°C, immobilized in AYE/0.5% agarose in 8-well ibidi dishes and let form microcolonies for 24 h at 30°C. After the addition of DPTA NONOate (0, 125, or 350 µM), P*_tac_-mCherry* and P*_flaA_-gfp* expression were recorded by time-lapse microscopy for 39 h, and microcolony formation was analyzed by 3D reconstruction (Imaris). Scale bars: 20 µm. The data shown are representative of three biological replicates.

The NO receptor genes localize to three different regions in the *L. pneumophila* genome, and each gene is organized in an operon (47, 50) (Fig. 1B). The gene *hnox1* forms an operon with *lpg1057*, *hnox2* with *lpg2458* and *lpg2457*, and *nosP* with *nahK* and *narR*, respectively. Using *gfp* fusion constructs to analyze the expression of the NO receptor gene promoters revealed that in the *L. pneumophila* parental strain JR32, P*_hnox1_* is expressed in the logarithmic growth phase showing a peak at the transition to the stationary growth phase, while P*_hnox2_* and P*_nosP_* are expressed to a lower extent and only in the stationary phase (Fig. 3A).

To assess the factors underlying the response of *L. pneumophila* to NO, we constructed and characterized marker-less mutant strains lacking individual (Hnox1, Hnox2, or NosP), two (double knockout, DKO), or all three (triple knockout, TKO) NO receptors. The NO receptor genes were deleted using a marker-less, non-polar knockout strategy (for details, see the Materials and Methods section), allowing not only to generate the single knockout strains (Δ*hnox1*, Δ*hnox2*, and Δ*nosP*) but also the double knockout (DKO, Δ*hnox1*-Δ*nosP*) and the triple knockout (TKO, Δ*hnox1*-Δ*nosP*-Δ*hnox2*) strain. The genomic deletions and lack of other mutations in the TKO mutant strain were verified by whole-genome sequencing (data not shown). The NO single and triple knockout receptor mutant strains grew indistinguishable from the parental strain in AYE broth or minimal defined medium, MDM (Fig. S3), and the TKO deletion mutant did not differ in the expression of the stationary phase promoters P*_flaA_* and P*_6SRNA_*, or the expression of the quorum sensing promoters P*_lqsA_* and P*_lqsR_* (Fig. S4). By contrast, the P*_lvbR_* promoter was expressed only in the Δ*nosP* and TKO mutant strains but neither in the JR32 parental strain nor in the Δ*hnox1* or Δ*hnox2* mutants (Fig. 3B). Taken together, compared to the parental strain, *L. pneumophila* single and triple NO receptor knockout strains grow indistinguishable in complex and defined media, and the expression of the *flaA*, *6SRNA*, *lqsA,* or *lqsR* genes is not affected in the TKO mutant strain, while *lvbR* is negatively regulated by NosP but neither by Hnox1 nor by Hnox2. Accordingly, NO-dependent regulation of LvbR specifically involves NosP (Fig. 1A).

### *L. pneumophila* lacking NO receptors is less responsive to chemically generated NO

To test whether the putative NO receptors Hnox1, Hnox2, or NosP mediate the response of *L. pneumophila* to NO, we exposed JR32 or the TKO mutant strain expressing P*_flaA_-gfp* to DPTA NONOate in AYE broth (Fig. 3C). Upon treatment with 400 µM DPTA, both *L. pneumophila* strains showed a delayed GFP production; however, DPTA NONOate reduced the production of GFP in the parental strain JR32, while the reduction of GFP production by DPTA NONOate was absent in the TKO mutant strain.

In another approach, we compared the effect of DPTA NONOate on the production of GFP under control of P*_flaA_* in sessile microcolonies formed by either the parental strain JR32 or the TKO mutant strain (Fig. 3D). Under these conditions, the treatment with 350 µM DPTA NONOate caused a delayed GFP production in the strain JR32, while the reduction of GFP production by DPTA NONOate was diminished in the TKO mutant strain, in particular at later time points (27–39 h). Taken together, these results indicate that the three NO receptors, Hnox1, Hnox2, and NosP, are indeed implicated in NO signaling in planktonic as well as sessile *L. pneumophila*.

### *L. pneumophila* NO receptor mutant strains are impaired for intracellular replication in macrophages and amoebae

**Fig 4:**
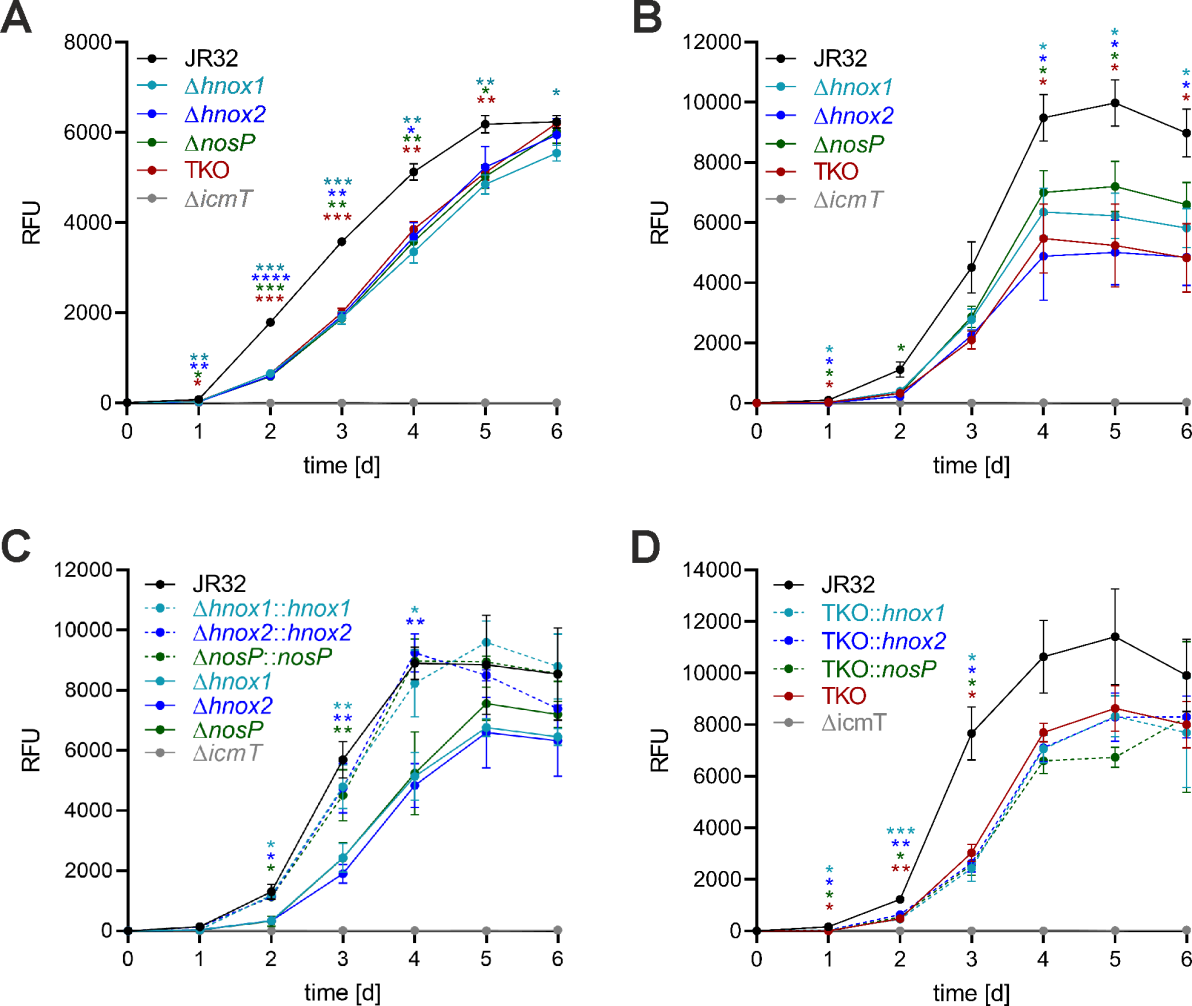
*L. pneumophila* NO receptor mutant strains are impaired for intracellular replication in macrophages and amoebae. (**A**) RAW 264.7 macrophages were infected (multiplicity of infection [MOI] 1, 6 days) with GFP-producing *L. pneumophila* JR32, Δ*icmT*, Δ*hnox1*, Δ*hnox2*, Δ*nosP,* or TKO mutant strains harboring pNT28. Intracellular replication was assessed by relative fluorescence units (RFU). *A. castellanii* amoebae were infected (MOI 1, 6 days) with GFP-producing *L. pneumophila* harboring pNT28: (**B**) strain JR32, Δ*icmT*, Δ*hnox1*, Δ*hnox2*, Δ*nosP,* or TKO, (**C**) strain JR32, the single NO receptor mutants or the complemented single mutants, or (**D**) strain JR32, the TKO mutant, or TKO with single NO receptor genes. Intracellular replication was assessed by GFP production and expressed as relative fluorescent units (RFU). Data shown are means and standard deviation of technical triplicates and representative of three biological replicates (*P   <  0.05, **P  <   0.01, ***P  <  0.001, ****P  <  0.0001; two-way ANOVA).

Next, we sought to assess whether the NO receptors are implicated in the virulence and host-pathogen interactions of *L. pneumophila*. To this end, we infected macrophages and amoebae with the parental strain or the single and triple NO receptor knockout strains. Upon infection of RAW 264.7 macrophages, the NO single knockout and TKO mutant strains grew significantly less efficiently compared to the parental strain JR32 (Fig. 4A). The mutant strains initiated growth later, followed by an apparently similar growth rate, and the growth defect of all mutants was comparable.

Similar results were obtained upon infection of the natural host amoeba, *A. castellanii*. The NO receptor single and triple knockout mutant strains grew significantly less efficiently compared to the parental strain JR32 (Fig. 4B). Upon growth in the amoebae, the initiation as well as the growth rate of the NO receptor mutant strains appeared to be impaired. The intracellular replication of the single mutant strains was restored to wild-type level upon re-introducing a single copy of the deleted gene into the genome of the mutant strains (Fig. 4C). Hence, the intracellular growth phenotype of the mutant strains is owed solely to the loss of the individual NO receptor genes and not due to second site or polar mutations. By contrast, re-introducing a single copy of the *hnox1*, *hnox2,* or *nosP* genes into the genome of the TKO mutant strain did not revert the intracellular growth defect of the mutant (Fig. 4D). These results indicate that the three different NO receptors, Hnox1, Hnox2, and NosP have non-redundant functions. In summary, *L. pneumophila* single NO receptor mutant strains and the TKO mutant are impaired for intracellular growth in macrophages and amoebae, the growth defect of the single mutants is complemented by re-introducing the corresponding gene into the mutant genome, and the TKO mutant cannot be complemented by single NO receptor genes.

### Intracellular growth heterogeneity of *L. pneumophila* is regulated by NO signaling

In eukaryotic host cells, the Lqs system regulates the size of the non-growing *L. pneumophila* persister subpopulation (28, 30) as well as the heterogeneous expression of the stationary growth phase promoter P*_flaA_* and LCV/host cell exit (30). To test whether the NO receptors and NO signaling affect the ratio of growing/non-growing intracellular *L. pneumophila*, we employed as a growth rate proxy the timer system, a stable fluorescent reporter protein that slowly maturates from a green to a red fluorescent form (28, 51).

**Fig 5:**
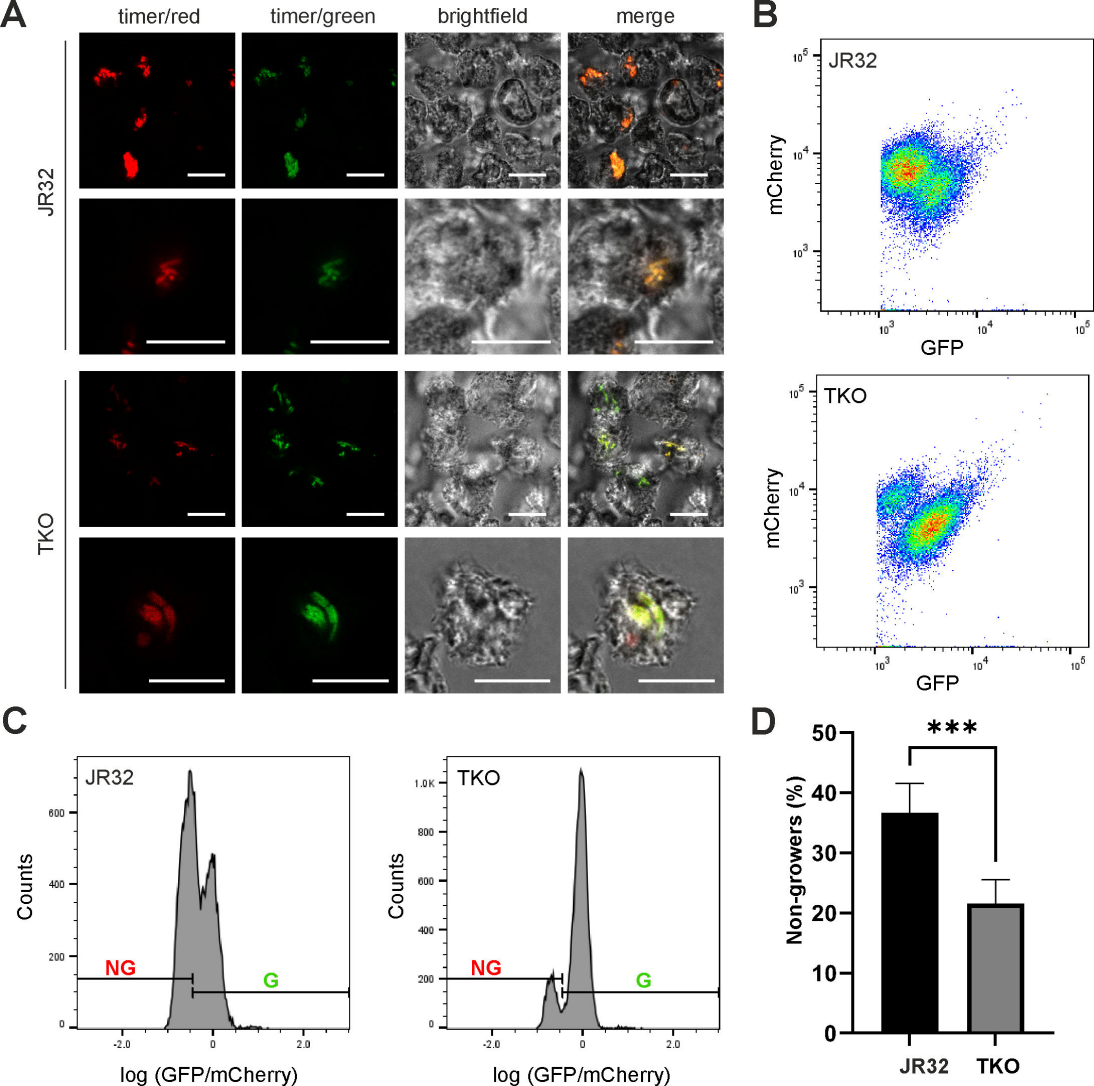
Phenotypic heterogeneity of *L. pneumophila* NO receptor mutant strains in amoebae. (**A**) *A. castellanii* amoebae were infected (multiplicity of infection 1, 20 h) with timer-producing *L. pneumophila* JR32 or TKO mutant strains harboring pNP107. The phenotypic heterogeneity was assessed (**A**) by confocal fluorescence microscopy in intact cells (scale bars, 10 µm), or (**B–D**) by flow cytometry in lysates. The flow cytometry data are depicted as (**B**) pseudocolor graph showing mCherry *versus* GFP signal intensity, (**C**) bacterial counts versus log (GFP/mCherry) to discriminate non-growing from growing *L. pneumophila*, and (**D**) bar graph depicting the percentage of non-growing bacteria. Data shown are representative of (**A**) two or (**D**) six experiments and show (**D**) means and standard deviations of six biological replicates (***, *P* < 0.001; Student’s *t*-test).

In initial experiments, *A. castellanii* amoebae were infected with timer-producing *L. pneumophila* JR32 or TKO mutant strains, and the ratio of growing (green) versus non-growing (red) intracellular bacteria was assessed by confocal fluorescence microscopy (Fig. 5A). Using this approach, a larger portion of the intracellular TKO mutant bacteria appeared green (growing), compared to parental strain JR32. This finding supports the notion that NO signaling positively regulates the emergence of non-growing intracellular *L. pneumophila*. The finding also suggests that the TKO mutant bacteria might reach the stationary growth phase later than JR32 bacteria, and thus, reflects the overall growth defect of the TKO mutant in macrophages (Fig. 4A) and *A. castellanii* (Fig. 4B).

To quantify the ratio of growing/non-growing intracellular *L. pneumophila*, we employed flow cytometry (Fig. 5B through D). *A. castellanii* amoebae were infected with timer-producing *L. pneumophila* JR32 or TKO mutant strains, and lysates of the infected amoebae were assessed for the intensity of GFP *versus* mCherry fluorescence (Fig. 5B) and bacterial counts *versus* log (GFP/mCherry) (Fig. 5C) to discriminate growing from non-growing *L. pneumophila*. This quantitative analysis revealed that compared to the JR32 parental strain, TKO mutant bacteria formed almost two times fewer non-growers in the amoebae (Fig. 5D), indicating that NO signaling indeed positively regulates the ratio of non-growing to growing *L. pneumophila*. Taken together, these results reveal that NO signaling regulates intracellular phenotypic heterogeneity of *L. pneumophila* and the ratio of non-growing *versus* growing bacteria in amoebae.

### *L. pneumophila* NO receptor mutant strains form mat-like biofilms

**Fig 6:**
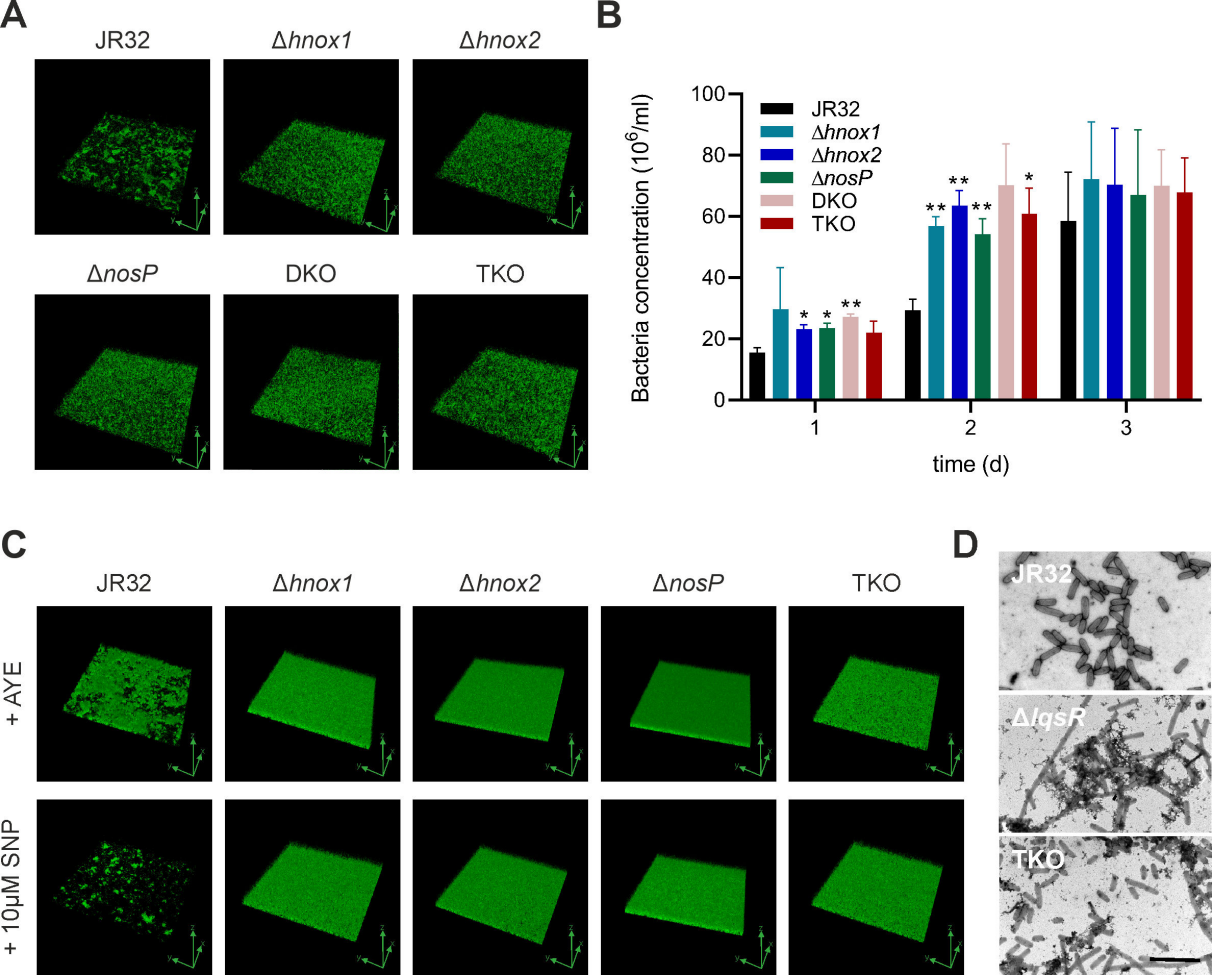
*L. pneumophila* NO receptor deletion strains form altered biofilms. Biofilms were initiated with exponential phase (18 h) GFP-producing *L. pneumophila* JR32, ∆*hnox1*, ∆*hnox2*, ∆*nosP*, or TKO mutant strains harboring pNT28 in ibiTreat microscopy dishes in AYE medium and grown at 25°C without mechanical disturbance for (**A**) 24 h or (**B**) 1–3 days. Confocal fluorescence microscopy images are shown as 3D representations (green axes: xyz orientation, dimensions: 185 × 185 × 30 µm). The images shown are representative of three independent experiments. The biomass of resuspended bacteria was determined by flow cytometry through the ratio of GFP-producing *L. pneumophila* (identified by fluorescence vs FSC) to beads population (identified by SSC vs FSC). Data shown are means and standard deviation (bacteria/mL) of biological triplicates from 100,000 events of each biofilm suspension (*P  <   0.05, **P  <   0.01; two-way ANOVA). (**C**) Biofilms formed for 24 h by *L. pneumophila* JR32, single NO receptor mutants, or the TKO mutant strain were treated or not with 10 µM SNP for another day. Dimensions (xyz): 185 × 185 × 30 µm. (**D**) Representative electron micrographs of *L. pneumophila* JR32, Δ*lqsR,* or TKO after 5 h sedimentation in AYE medium. The samples were mounted on copper grids and stained with uranyl formate. Scale bar, 5 µm.

To assess the role of the NO receptors for *L. pneumophila* biofilm formation, GFP-producing *L. pneumophila* JR32 or ∆*hnox1*, ∆*hnox2*, ∆*nosP*, DKO, or TKO mutant strains were seeded onto ibidi µ-dishes and left to form biofilms in AYE medium for 1, 2, and 3 days at 25°C without disturbance. Biofilm formation, including architecture and adherence to an abiotic surface, was analyzed by confocal microscopy (Fig. 6).

Under these conditions, the attachment of the parental JR32 strain and the NO receptor mutant strains to the abiotic dish surface was undistinguishable (Fig. S5A). However, the biofilm formed by the JR32 strain showed a “clustered” and “patchy” appearance, while the ∆*hnox1*, ∆*hnox2*, ∆*nosP*, DKO, or TKO mutant strains formed a “mat-like” biofilm with a different 2D- and 3D architecture (Fig. 6A; Fig. S5B). Quantification of the biofilm mass by flow cytometry revealed that the biomass of the NO receptor mutant strain biofilms was larger than that of the parental strain at early times during biofilm formation (Fig. 6B); yet, high-resolution scanning electron microscopy (SEM) images of wild-type and TKO biofilms were similar (Fig. S5C). In summary, biofilms formed by *L. pneumophila* lacking one (Hnox1, Hnox2, and NosP), two (DKO), or all three (TKO) NO receptors adopt a “mat-like” architecture morphologically distinct from the “patchy” morphology of biofilms formed by the parental strain JR32, and the mutant biofilms contain more biomass.

We also tested whether NO signaling affects the dispersal of *L. pneumophila* biofilms. To this end, we compared the effect of the chemical NO donor SNP on the dispersal of 1-day-old biofilms formed by GFP-producing *L. pneumophila* JR32, single NO receptor or TKO mutant strains (Fig. 6C). Intriguingly, 10 µM SNP promoted the dispersal of a biofilm formed by the parental strain JR32 but not biofilms formed by single NO receptor or TKO mutant bacteria. These findings indicate a role of the NO receptors Hnox1, Hnox2, and NosP for NO signaling and dispersal of *L. pneumophila* biofilms and confirm a non-redundant function of the three NO receptors.

Biofilms are encased by a complex extracellular matrix. To assess whether NO signaling plays a role in the formation of extracellular structures, we performed sedimentation experiments, which previously revealed the enhanced formation of extracellular filaments by the *L. pneumophila* Δ*lqsR* and Δ*lqsS* mutant strains (31, 32). *L. pneumophila* JR32, single NO receptor mutants, TKO, and Δ*lqsR* mutant strains were grown on CYE agar plates, gently suspended in AYE medium, and let sediment by gravity for 4 h at room temperature (Fig. S6A). Quantification of the sedimentation rate revealed that single NO receptor mutants sedimented like the parental JR32 strain (data not shown), while the TKO mutant strain sedimented significantly slower than JR32, albeit still faster than the Δ*lqsR* mutant (Fig. S6B). A high-resolution analysis by EM indicated that the TKO mutant formed a more extensive network of extracellular filaments (Fig. 6D), and therefore, the sedimentation rate of the *L. pneumophila* JR32, TKO, and Δ*lqsR* mutant strains is inversely correlated to the formation of extracellular filaments.

## DISCUSSION

In this study, we investigated the response of *L. pneumophila* to NO and characterized the bacterial receptors underlying this process. We found that the chemical NO donors SNP and DPTA NONOate delayed and reduced the expression of the promoters for flagellin (P*_flaA_*) and the 6S small regulatory RNA (P*_6SRNA_*) (Fig. 2). Compared to the parental *L. pneumophila* strain JR32, P*_flaA_* expression was initially much less pronounced and delayed in the presence of DPTA NONOate in the TKO strain, indicating that the NO receptors are implicated in NO signaling *in vivo* (Fig. 3). Moreover, the *L. pneumophila* single and triple NO receptor mutant strains were impaired for growth in macrophages and amoebae (Fig. 4) and the NO receptors regulated the intracellular phenotypic heterogeneity of *L. pneumophila* such that the non-growing subpopulation was larger in the parental strain compared to strains lacking the NO receptors (Fig. 5). Finally, the single and triple NO receptor mutant strains showed a mat-like biofilm architecture, and NO-dependent biofilm dispersal was dependent on the NO receptors (Fig. 6).

NO is a highly reactive free radical, has a lifetime of only a few seconds, and diffuses freely across membranes. These characteristics render NO ideal for short-range and transient signaling within and between neighboring cells (44, 45). Accordingly, NO signaling is implicated in the intracellular replication of *L. pneumophila* in phagocytes (Fig. 4) as well as in biofilm dynamics (Fig. 6). These findings are consistent with the idea that NO signaling in the micrometer range is important for pathogen-host cell interactions as well as for bacterial cell-cell communication.

The NO receptor gene promoters P*_hnox1_*, P*_hnox2_*, and P*_nosP_* are differentially expressed upon growth of *L. pneumophila* in broth (Fig. 3A), and *L. pneumophila* mutant strains lacking individual NO receptors all showed a similar defect in intracellular replication in macrophages and amoebae (Fig. 4A and B). These findings suggest that the three different NO receptors, Hnox1, Hnox2, and NosP, are non-redundant and might adopt specific functions during growth in media as well as during intracellular growth. Rather surprisingly, the TKO mutant also did not show a more severe intracellular growth phenotype than the three single NO receptor mutant strains. In agreement with a non-redundant function of the individual NO receptors, the TKO mutant strain was not complemented by the genomic integration of individual NO receptor genes (Fig. 4D). Furthermore, the single as well as the TKO mutant strains formed architecturally altered biofilms, which no longer dispersed following the administration of NO (Fig. 6). Accordingly, all three NO receptors are required for the response of *L. pneumophila* biofilms to NO, supporting the notion of non-redundant functions of the NO receptors.

Possible explanations for the observed non-redundancy of the three NO receptors are that the receptors obligatorily crosstalk among each other, and/or, they do not trigger the same downstream signaling pathway(s) regulating different features of *L. pneumophila*. As an example, NosP but neither Hnox1 nor Hnox2 negatively regulates the transcription factor LvbR (Fig. 3B). While additional pathways specific for an individual NO receptor are likely in operation, the second messenger c-di-GMP is a candidate for convergent signaling through the three NO receptors. The Hnox-1 (42) and NosP (46) signaling pathways have experimentally been shown to converge on modulating c-di-GMP; yet, for Hnox-2, the downstream second messenger(s) or signaling cascade is not known. In agreement with the important role of c-di-GMP signaling for *L. pneumophila*-host cell interactions, several proteins predicted to contain domains related to c-di-GMP synthesis, hydrolysis, or recognition were found to modulate intracellular growth of *L. pneumophila* (52, 53).

Intriguingly, NO signaling regulates intracellular phenotypic heterogeneity of *L. pneumophila* by modulating the ratio of growing to non-growing bacteria (Fig. 5). Phenotypic heterogeneity underlies the formation of a virulent and motile *L. pneumophila* subpopulation, which preferentially exits the LCV and the amoeba host cell, and thus, seems to engage in “division of labor” (30). The Lqs quorum sensing system and the LAI-1 signaling molecule modulate these aspects of phenotypic heterogeneity (28, 30). We show here that in addition to quorum sensing, NO signaling also orchestrates intracellular phenotypic heterogeneity of *L. pneumophila*, and thus, governs short-distance division of labor of the pathogen.

Bacterial cell-cell communication in sessile communities also requires signaling in the µm range. Accordingly, NO signaling occurs during microcolony formation (Fig. 3D) and is implicated in biofilm formation and dispersal of *L. pneumophila* (Fig. 6). The TKO mutant strain showed a “mat-like” rather than a “patchy” biofilm architecture (Fig. 6A and C), and single NO receptor mutant or TKO biofilms no longer respond to NO (Fig. 6C). NO is commonly involved in the regulation of bacterial biofilms (45). Biofilm formation is frequently regulated by NO-binding H-NOX domain proteins and downstream signaling networks such as two-component systems or c-di-GMP metabolism (54). Accordingly, several c-di-GMP metabolizing enzymes are involved in the regulation of *L. pneumophila* biofilm formation (42, 43). Specifically, the DGC Lpg1057/Lpl1054 has been implicated in NO-mediated c-di-GMP production (Fig. 1A) and its effects on biofilms (43).

The pleiotropic transcription factor LvbR regulates the architecture of *L. pneumophila* biofilms such that the Δ*lvbR* mutant strain forms a “mat-like” biofilm (41), similar to the single NO receptor and TKO mutant strains (Fig. 6). NosP negatively regulates LvbR (Fig. 3B), apparently conflicting with the similar biofilm phenotypes of the corresponding mutant strains. LvbR- and NO-mediated signaling pathways seem to be complex, and downstream targets might involve components of c-di-GMP signaling. LvbR (41) and NosP (46) positively regulate c-di-GMP signaling through Lpg1057 or NarR, respectively (Fig. 1A). Perhaps, under the conditions tested, these regulatory pathways are dominant over the negative regulation of LvbR by NosP. LvbR also downregulates flagellar genes in biofilms (41), and the chemical NO donors SNP and DPTA NONOate delayed and reduced the expression of the flagellin promoter (P*_flaA_*) in planktonic and sessile *L. pneumophila* (Fig. 2 and 3). However, an *L. pneumophila* Δ*flaA* mutant strain forms “patchy” biofilms like the parental strain (41), and therefore, the flagellum does not account for the altered biofilm architecture of Δ*lvbR* and TKO mutant biofilms.

Beyond a similar biofilm morphology of *L. pneumophila* strains lacking the NO receptors or *lvbR*, there appears to be an intimate link between NO signaling and LvbR. On a transcriptional level, LvbR is a negative regulator of Hnox1, and consequently, reduces NO signaling. Reciprocally, the P*_lvbR_* promoter is induced in the absence of NO signaling in the Δ*nosP* and TKO mutant strains (Fig. 3B) but not in the parental strain JR32, while several other promoters (P*_flaA_*, P*_6SRNA_*, P*_lqsA_*, and P*_lqsR_*) are not differentially regulated (Fig. S4). Since NosP-mediated signaling negatively regulates the Hnox1 inhibitor LvbR, NO signaling appears to be amplified by a positive feedback loop. However, this regulatory loop might not occur in all *L. pneumophila* strains, as P*_lvbR_* was transiently expressed in several clinical and environmental *L. pneumophila* isolates (33).

The Lqs system regulates NO signaling through LvbR (40) (Fig. 1A), since the sensor kinase LqsS negatively regulates LvbR, which, in turn, negatively regulates Hnox1 (32, 41). Accordingly, the Lqs system is a positive regulator of NO signaling in *L. pneumophila*, representing a prototypical and novel regulation of NO signaling by quorum sensing. In *L. pneumophila*, NO regulates motility (P*_flaA_*) dependent on the NO receptors (Fig. 2 and 3), but the NO receptors do not seem to regulate stress response (P*_6SRNA_*) and components of the Lqs system (P*_lqsA_*, P*_lqsR_*) (Fig. S4). While in *L. pneumophila* quorum sensing regulates NO signaling, NO signaling regulates quorum sensing in other bacteria (55).

NO signaling has been found to regulate quorum sensing in different bacteria, including pathogens of the genera *Vibrio* and *Pseudomonas* (55). *Vibrio harveyi* shows a NO-dependent increase in bioluminescence, which is relayed through the pivotal quorum sensing histidine phosphotransfer protein LuxU (56). The binding of NO to the sensor H-NOX inhibits the cytosolic hybrid histidine kinase HqsK, which, in turn, leads to dephosphorylation of the pivotal integrator of quorums sensing, LuxU, in *V. harveyi* (56) as well as in *Vibrio parahaemolyticus* (57). Analogously, in *Vibrio cholerae*, binding of NO to the sensor NosP inhibits the cytosolic hybrid histidine kinase VpsS, which decreases the levels of phosphorylated LuxU (58). Intriguingly, the effect of NO is only observed at low cell density, when the other three quorum sensing pathways converging on LuxU are not operational (56). NO has also been reported to reduce flagellum production and to enhance biofilm formation in an H-NOX-dependent manner in *V. harveyi* (59), while leading to biofilm dispersal of *V. cholerae* (49) and *Vibrio fischeri* (60). Overall, the crosstalk between quorum sensing and NO signaling regulates virulence and biofilm formation in a number of human pathogens, including the genera *Legionella*, *Vibrio,* and *Pseudomonas*.

In summary, we demonstrate in this study that *L. pneumophila* regulates virulence, intracellular phenotypic heterogeneity, and biofilm dynamics through NO and the three NO receptors, Hnox1, Hnox2, and NosP. Further studies will identify and characterize the molecular components underlying NO-dependent *L. pneumophila*-phagocyte inter-kingdom signaling, biofilm architecture, biofilm dynamics and phenotypic heterogeneity.

## MATERIALS AND METHODS

### Bacteria, eukaryotic cells, and growth conditions

*L. pneumophila* strains (Table 1) were grown on CYE agar plates at 37°C for 2–4 days (61) or in liquid cultures with *N*-(2-acetamido)-2-aminoethanesulfonic acid (ACES)-buffered yeast extract (AYE) medium (62) for 18–22 h on a wheel (80 rpm). AYE medium was supplemented with chloramphenicol (Cm; 5 µg/mL) to maintain plasmids if required.

**TABLE 1:** Bacterial strains and plasmids used in this study

Strain or plasmid	Relevant properties^*a*^	Reference
*E. coli*		
TOP10		Invitrogen
DH5α λpir	Producing Pir protein for replication via *oriR6K*	(63, 64)
*L. pneumophila*		
GS3011 (Δ*icmT*)	JR32 *icmT3011*::Km	(65)
JR32	*L. pneumophila* Philadelphia-1, serogroup 1, a salt-sensitive isolate of AM511	(66)
NT03 (Δ*lqsR*)	JR32 *lqsR*::Km	(34)
SM01 (Δ*hnox1*)	JR32 *hnox1* markerless deletion	This study
SM02 (Δ*nosP*)	JR32 *nosP* markerless deletion	This study
SM03 (Δ*hnox2*)	JR32 *hnox2* markerless deletion	This study
SM04 (DKO)	JR32 *hnox1*, *nosP* markerless double knockout	This study
SM05 (TKO)	JR32 *hnox1*, *nosP*, *hnox2* markerless triple knockout	This study
Plasmids		
pCM009	pMMB207C-P*_flaA_-gfp* (ASV), Cm	(27)
pCS031	pMMB207C-P*_hnox2_-gfp* (ASV), Cm	This study
pCS032	pMMB207C-P*_nosP_-gfp* (ASV), Cm	This study
pNP102	pMMB207C, Δ*lacI*^q^, P*_tac_-mcherry* (const.), Cm	(67)
pNP107	pMMB207C, Δ*lacI*^q^, P*_tac_-timer* (const.), Cm	(28)
pNT28	pMMB207C, Δ*lacI*^q^, P*_tac_-gfp* (const.), Cm	(34)
pNT31	pMMB207C, Δ*lacI*^q^, P*_tac_-gfp* (const.), P*_lqsS_-lqsS*	(32)
pRH023	pMMB207C-P*_lvbR_-gfp* (ASV), Cm	(41)
pRH026	pMMB207C-P*_hnox1_-gfp* (ASV), Cm	(41)
pRH035	pMMB207C-P*_sidC_-gfp* (ASV), Cm	(30)
pRH037	pMMB207C-P*_lqsR_-gfp* (ASV), Cm	(33)
pRH038	pMMB207C-P*_lqsA_-gfp* (ASV), Cm	(33)
pRH049	pMMB207C-P*_6SRNA_-gfp* (ASV), Cm	(24)
pSM015	pMMB207C, Δ*lacI*^q^, P*_tac_-gfp* (const.), P*_hnox1_-hnox1*	This study
pSM016	pMMB207C, Δ*lacI*^q^, P*_tac_-gfp* (const.), P*_nosP_-nosP*	This study
pSM017	pMMB207C, Δ*lacI*^q^, P*_tac_-gfp* (const.), P*_hnox2_-hnox2*	This study
pSM028	pSR47S-flanking regions_*hnox1*	This study
pSM031	pSR47S-flanking regions_*nosP*	This study
pSM032	pSR47S-flanking regions_*hnox2*	This study
pSM036	pSR47S-*hnox1*-flanking regions_*hnox1*	This study
pSM037	pSR47S-*nosP*-flanking regions_*nosP*	This study
pSM038	pSR47S-*hnox2*-flanking regions_*hnox2*	This study
pSN7	pMMB207C, Δ*lacI*^q^ (truncated), P*_tac_*-RBS_T7_-*mcherry* (const.), P*_flaA_-gfp* (AAV), Cm	(28)
pSR47S	*oriR6K*, *sacB*, Km	(63, 64)

^*a*^ Abbreviations: Cm, chloramphenicol resistance; Km, kanamycin resistance; const., constitutive.

To determine growth characteristics, *L. pneumophila* JR32 and the Δ*hnox1*, Δ*hnox2*, Δ*nosP*, the DKO, or the TKO mutant strains were grown for 18 h at 37°C in AYE medium, diluted to an initial OD_600_ of 0.2 in a black clear bottom 96-well plate (100 µL/well) and then incubated at 30°C while orbitally shaking. Growth was monitored in triplicates by measuring the absorbance at 600 nm (OD_600_) in a microtiter plate reader (Synergy H1 Hybrid Reader BioTek, or Cytation 5 Hybrid Multi-Mode Reader, Agilent Technologies). Several independent Δ*hnox1*, Δ*hnox2*, Δ*nosP*, and TKO clones were tested.

*A. castellanii* (ATCC: 30234, lab collection) was cultured in proteose, yeast extract, glucose (PYG) medium (68, 69) at 23°C using proteose from Becton Dickinson Biosciences and yeast extract from Difco. Murine RAW 264.7 macrophages (ATCC: TIB-71, lab collection) were grown in RPMI 1640 medium (Life Technologies) supplemented with 10% fetal calf serum (FCS; Life Technologies) and 1% L-glutamine (Life Technologies) in a humidified atmosphere at 37°C in 5% CO_2_.

### Molecular cloning and generation of *L. pneumophila* scar-free deletion mutants

Cloning was performed according to standard protocols, and plasmids were isolated using the NucleoSpin plasmid kit (Macherey-Nagel). DNA fragments were amplified using Q5 (NEB) or Phusion (Thermo scientific) high-fidelity DNA polymerase. DNA fragments were assembled *via* Gibson assembly using the NEBuilder HiFi DNA assembly kit (NEB). DNA fragments were amplified using the primers listed in Table S1, and the plasmids used in this study are listed in Table 1. All constructs were verified by DNA sequencing.

The scar-free single or multiple NO receptor deletion mutants were generated by double homologous recombination. The genes of interest as well as 1 kb of their upstream and downstream region were cloned into the suicide plasmid pSR47S (63, 64), and the relevant genes were deleted except for overlapping regions with adjacent genes in the case of *hnox1* and *hnox2*. For the construction of suicide plasmids, the gene of interest and ca. 1 kb of their upstream and downstream region were amplified using the primer pairs oSM124/oSM125 for *hnox1* (1154,000–1,154,542 bp), oSM130/oSM131 for *nosP* (332,290–333,456 bp), or oSM134/oSM135 for *hnox2* (2,773,093–2,773,653 bp). The individual amplified fragments were then assembled with linearized pSR47S (generated by amplification using oSM113/oSM114) to yield pSM036, pSM037, and pSM038. These intermediate vectors were also used for the complementation by chromosomal reintegration of the NO receptor genes at the original site in the genome. The gene of interest was then deleted using vector linearization PCR with the primer pairs oSM126/oSM127 for Δ*hnox1*, oSM130/oSM131 for Δ*nosP,* and oSM136/oSM137 for Δ*hnox2*, yielding pSM028, pSM031, and pSM032. In the case of *hnox1* and *hnox2*, overlapping regions with *lpg1057* or *lpg2458*, respectively, were not deleted and kept in the upstream fragments.

*L. pneumophila* JR32 was transformed with plasmid pSM028 for *hnox1*, pSM031 for *nosP,* and pSM032 for *hnox2* deletion, yielding the strains SM01, SM02, and SM03. Co-integrates were selected on CYE plates supplemented with kanamycin (Km; 20 µg/mL). Single colonies were picked and streaked on CYE plates to allow for a second recombination event, followed by streaking single colonies on freshly prepared CYE plates supplemented with 5%-10% sucrose. Single colonies were then patched on CYE with and without Km plates. The correct genomic deletion in Km-sensitive clones was verified by colony PCR and sequencing. The TKO was generated by transforming Δ*hnox1* with pSM031 and performing the selection process to yield a double knockout mutant Δ*hnox1*-Δ*nosP* (SM04), which subsequently was transformed with pSM032 and subjected to the selection process a third time to yield the TKO strain (SM05). The genomic deletions of the TKO mutant strain and lack of other mutations were validated by whole-genome sequencing.

Plasmids pCS031 and pCS032 harboring a transcriptional P*_hnox2_*- or P*_nosP_-gfp* fusion were constructed by PCR amplification of the promoters for *hnox2* (200 bp) or *nosP* (200 bp) using the primer pair oCS103/104 or oCS105/106 and cloned into the SacI and XbaI sites of pCM009 (27) using the NEBuilder HiFi DNA assembly reaction.

To construct the plasmids pSM015, pSM016, and pSM017 harboring *hnox1*, *nosP*, or *hnox2* under the control of their promoters, the corresponding gene regions were amplified together with their putative native promoter regions (600 bp upstream) using Phusion polymerase, JR32 genomic DNA as a template, and the primer pairs oSM059/oSM060, oSM061/oSM062, and oSM063/oSM064, respectively. The PCR products were purified and cloned into pNT31 (constitutively expressing *gfp*), previously digested with BamH1 and Xho1. These plasmids were used for complementation assays but did not restore the phenotype of the parental strain JR32.

Bacterial growth and GFP production were monitored in triplicates by measuring the absorbance at 600 nm (OD_600_) and fluorescence (excitation, 485 nm; emission, 528 nm; gain, 50) using a microtiter plate reader. Promoter activity was inferred by *gfp* expression levels, denoted as relative fluorescence units divided by log_10_(bacterial counts).

### Expression of GFP reporter constructs in *L. pneumophila*

Promoter expression was assessed by a plate reader and quantified by flow cytometry as described (33). Briefly, *L. pneumophila* strains (JR32, ∆*hnox1*, ∆*hnox2*, ∆*nosP,* or TKO) harboring P*_flaA_*-, P*_6SRNA_*-, P*_sidC_*-, P*_hnox1_-,* P*_hnox2_-,* P*_nosP_-,* P*_lvbR_*-, P*_lqsR_*-, or P*_lqsA_-gfp* (ASV) fusion reporter plasmids (pCM009, pRH049, pRH035, pRH026, pCS031, pCS032, pRH023, pRH037, or pRH038) were grown for 3 days on CYE/Cm agar plates followed by growth in AYE/Cm (5 µg/mL) on a rotating wheel (80 rpm) at 37°C for 18 h. On the day of the experiment, fresh stock solutions and a dilution series of the chemical NO generators were prepared. For SNP, a 100 mM stock solution was prepared directly in AYE medium. For DPTA NONOate, 40 mM stock solutions were prepared, either in PBS pH 7.4 or in PBS pH 5.0 (for spent DPTA NONOate), adjusted by adding HCl. As DTPA NONOate is sensitive to moisture and should be kept sealed, a new 10 mg vial was used for each experiment. At the concentrations used (5 µM SNP, 400 µM DPTA NONOate), we did not observe the effects of the pharmacological NO donors on the survival of *L. pneumophila* but there was a slightly prolonged lag phase.

Bacterial strains from overnight cultures were subsequently inoculated in a black, clear bottom 96-well plate (100 mL/well, polystyrene) at an initial OD_600_ of 0.2 in AYE/Cm (5 µg/mL) supplemented or not with the NO generators to reach the final concentrations indicated for SNP (0, 2.5 or 5 µM), DPTA NONOate (0, 250, 300 or 400 µM), or spent DPTA NONOate (0 or 400 µM), and the strains were incubated at 30°C while orbitally shaking. Bacterial growth and GFP production were monitored in triplicates by measuring the OD_600_ and fluorescence (excitation, 485 nm; emission, 528 nm; gain, 50) with a microplate reader. Blank values (AYE medium) were subtracted from all samples and promoter activity was inferred by *gfp* expression levels, denoted as relative fluorescence units (RFU) divided by log_10_(bacterial counts).

To quantify promoter expression by flow cytometry, *L. pneumophila* JR32 strain harboring P*_flaA_*- or P*_6SRNA_-gfp* (ASV) fusion reporter plasmids (pCM009, or pRH049) were grown at 37°C in AYE/Cm (5 µg/mL) supplemented with SNP to reach a final concentration of 0, 2.5, and 5 µM SNP from a freshly prepared 100 mM stock solution in AYE. Every 2 h from 18 to 28 h, the bacteria were fixed with 4% paraformaldehyde (PFA) for 1 h at room temperature (RT) and subsequently stained with 1 µg/mL DAPI for 45 min at RT in the dark. The GFP-positive population was quantified using a Fortessa II flow cytometer and Diva software. The bacterial population was identified employing a forward (FSC, 650 V) and sideward scatter (SSC, 240 V) gating, with a threshold of 200 each, examined for DAPI stain (Vio 450_50, 700 V) and GFP production (Blue 530_30, 600 V), and 10,000 events per sample were recorded. The data were analyzed with the software FlowJo. The GFP-positive population was gated using a GFP control and a DAPI control as reference. The strain JR32/pNT028, constitutively producing GFP, served as the GFP control, and the parental JR32 strain as the DAPI control.

### Microcolony growth

Microcolony growth of *L. pneumophila* and bacterial GFP production was assessed as described (29). Briefly, *L. pneumophila* strains (JR32, TKO) carrying the dual reporter P*_tac_-mcherry*/P*_flaA_-gfp* (pSN7) were grown on CYE agar plates supplemented with Cm (5 µg/mL) at 37°C for 2 days. Bacterial lawns were used to inoculate AYE medium/Cm (5 µg/mL) at an OD_600_ of 0.1 and grown on a wheel (80 rpm) for ca. 21–22 h. Stationary phase bacteria were embedded in AYE medium/0.5% agarose/Cm (5 µg/mL) at an OD_600_ of 0.1 and poured into chambered coverslips (ibidi 8-well μ-slide). After solidification, bacteria were allowed to grow overnight at 30°C to form microcolonies. The next day, DPTA NONOate was freshly dissolved in PBS and added to the wells at the final concentrations indicated (0, 125, 175, or 350 µM). Microcolony formation was monitored by confocal microscopy at 30°C. To generate 3D images, 100–200 slices were recorded.

### Biofilm formation and dispersal

The formation and quantification of biofilm by *L. pneumophila* was determined as described previously (33, 41). Briefly, GFP-producing *L. pneumophila* strains (pNT28) were grown for 3 days on CYE/Cm agar plates, followed by growth in AYE/Cm on a rotating wheel (80 rpm) at 37°C for 18 h. The bacteria were diluted to an OD_600_ of 0.3 in AYE/Cm, 2 mL were placed into an ibiTreat microscopy dish (ibidi), and incubated at 25°C for 24 h while avoiding mechanical disturbance. The biofilm architecture was monitored by confocal fluorescence microscopy (Leica TCS SP8 X CLSM, objective: HC PL APO CS2, 63 x/1.4–0.60 oil; Leica Microsystems) by acquiring images at the dish bottom (0 µm, attachment layer) or at a level of 4 µm above the dish bottom and the 3D-images were generated using ImageJ. To assess biofilm dispersal upon the addition of NO, a final concentration of 10 µM SNP dissolved in 1 mL AYE was carefully added to 1-day-old biofilms. Biofilms were then incubated for another day at 25°C before images were acquired by confocal fluorescence microscopy as described.

Biofilm formation was quantified as described (33). Briefly, GFP-producing *L. pneumophila* strains (pNT28) were left to form biofilms at 25°C for 3 days. The biofilms were resuspended by excessive pipetting, transferred into a falcon tube, and excessively vortexed, and the OD_600_ was determined (usually 3.3–4.1). Subsequently, the suspension was diluted 1:100 (2 µL beads:200 µL bacteria) with a well-mixed microsphere standard suspension (Bacteria Counting Kit, Invitrogen). The biofilm mass (bacteria/mL, 10^5^ events) was quantified using a Fortessa II flow cytometer and Diva software. Before measuring, samples were vortexed for 8 s. Bacteria and microsphere standards were determined employing a forward (FSC, 650V) and sideward scatter (SSC, 230V), with a threshold of 200 each, *L. pneumophila* was identified by GFP production (Blue 530_30: 350V). The data were analyzed with the software FlowJo. The gating protocol was as follows: beads were identified using a control sample (AYE/Cm without bacteria) supplemented with beads as a reference (gate 1, FSC vs SSC). The number of events counted in the microsphere gate provides an accurate estimate of the volume analyzed. The *L. pneumophila* populations were depicted in pseudocolor graphs (gate 2, GFP vs FSC) using a resuspended biofilm sample (of strain JR32) without beads as a reference. Bacterial density in the biofilm was determined from the ratio of *L. pneumophila* (gate 2) to beads (gate 1).

The *L. pneumophila* biofilm structure was assessed by SEM (32). One-day-old biofilms were fixed with glutaraldeyde (2.5%) in cacodylate buffer, followed by treatment with Dulbecco’s PBS (pH 7.35), 1% osmium, Dulbecco’s PBS (pH 7.35), 70% ethanol, 100% ethanol, and hexamethyldisilazane. After drying, the samples were covered with platinum and imaged by SEM.

### Sedimentation and formation of extracellular filaments

The sedimentation behavior of different *L. pneumophila* strains was assessed as published (31, 32). Briefly, to analyze sedimentation, *L. pneumophila* strains (JR32, TKO, and Δ*lqsR*) constitutively producing GFP (pNT28) were grown for 4 days on CYE agar plates, gently (without vortexing) suspended in AYE medium to a final OD_600_ of 5.0 and let sediment by gravity for 4–6 h at RT. The sedimentation rate was visualized by recording the bacterial fluorescence using a UV lamp and quantified as mm per h.

The appearance of the *L. pneumophila* strains during sedimentation was also assessed by EM (32). To this end, samples were taken after 5 h of sedimentation, mounted on copper grids, stained with uranyl formate, and viewed with the FEI G2 Tecnai spirit electron microscope.

### Intracellular replication of *L. pneumophila* in macrophages

Intracellular replication of *L. pneumophila* in macrophages was determined as published (28, 67). Briefly, RAW 264.7 macrophages were cultivated in the supplemented RPMI 1640 medium, diluted the day before infection, and seeded in 96-well plates (7 × 10^4^ cells per well). GFP-producing *L. pneumophila* strains (pNT28) were inoculated at an OD_600_ of 0.1 in AYE/Cm (5 µg/mL), grown to early stationary phase at 37°C, and diluted in supplemented RPMI to yield a final multiplicity of infection (MOI) of 1. The infection was synchronized by centrifugation (400 × *g*, 10 min, RT). After 1.5 h, the infected cells were washed with supplemented RPMI medium, the plates were incubated at 37°C with 5% CO_2_ in a humidified atmosphere, and intracellular bacterial replication was assessed by measuring GFP production with a plate reader.

### Intracellular replication of *L. pneumophila* in *A. castellanii*

Intracellular replication of *L. pneumophila* in *A. castellani* was determined as published (41). Briefly, the amoebae were seeded in PYG in a black clear bottom 96-well plate (2 × 10^4^ cells/well) and left to adhere at 30°C for 24 h (cell numbers approximately doubled in this time). The media and non-adherent amoebae were aspirated, and adherent amoebae were washed with Ac buffer. GFP-producing *L. pneumophila* (pNT28) was grown in AYE media supplemented with Cm (5 µg/mL) for 21–22 h at 37°C to early stationary phase (OD_600_ ca. 5, ~2 × 10^9^ bacteria/mL). The cultures were routinely checked under the microscope (motile, non-filamentous bacteria). Cultures were diluted in Ac buffer to the desired density and used to infect the amoebae at an MOI of 1. The infection was synchronized by centrifugation (400 × *g*, 10 min, RT) and then incubated at 30°C. Intracellular bacterial replication was assessed by measuring GFP production with a plate reader.

To assess phenotypic heterogeneity, *A. castellanii* amoebae were infected with *L. pneumophila* JR32 or TKO carrying pNP107 as described above, except that culture-treated 6-well plates (VWR) were used with 8.5 × 10^5^ amoebae seeded per well. The amoebae were infected at an MOI of 1, centrifuged (1,000 × *g*, 10 min, RT), and incubated at 30°C for 24 h. For visualization of phenotypic heterogeneity by confocal fluorescence microscopy (Leica SP8 inverse, 63 × oil objective), the infected amoebae were collected and fixed with 4% PFA (1 h), washed twice with PBS, transferred to an 18-well µ-slide dish (Ibidi), and immobilized by adding a layer of PBS/0.5% agarose.

For flow cytometry analysis of phenotypic heterogeneity, the infected amoebae were collected, lysed with 0.1% Triton TX-100 (Sigma) in 150 mM NaCl (30 min, RT), pelleted, fixed with 4% PFA (1 h), washed twice with PBS, and finally collected in 0.5 mL PBS and stored at 4°C until use. A Fortessa II flow cytometer and Diva software were used to record the relevant spectral parameters. Bacterial populations were identified employing a forward (FSC, 650 V) and sideward scatter (SSC, 300 V) gating, each with a threshold of 200. The bacterial population was further examined for GFP (Blue 530_30, 700 V) and mCherry production (YG 610/20, 700 V). Approximately 2 × 10^4^ GFP-positive bacteria per sample were recorded. The data were analyzed using FlowJo software. The gating protocol was as follows: (i) The bacterial population was defined in an SSC-H and FSC-H pseudocolor graph, (ii) GFP-positive bacteria were determined in a GFP-H vs FSC-H graph, and (iii) the spectral properties collected using the Timer reporter were analyzed by calculating log(<Param name=”Blue 530_30 H”/>/<Param name= “YG 610_20 H”/>) and defining a growers/non-growers gate in a histogram.

### Statistics

Statistics were determined by a Student’s *t*-test or two-way ANOVA on the means and standard deviations of three replicates, using untreated bacteria as reference values in pharmacological experiments, or compared to *L. pneumophila* JR32-infected host cells in infection experiments. The statistical analysis was performed using the GraphPad Prism (Version 9.5.1) software, and differences were deemed statistically significant when the *P*-value was less than 0.05.

## Data Availability

All data are contained within the paper.

## SUPPLEMENTAL MATERIAL

Supplemental material. mbio.00710-24-s0001.pdf Fig. S1-S6; Table S1.

ASM does not own the copyrights to Supplemental Material that may be linked to, or accessed through, an article. The authors have granted ASM a non-exclusive, world-wide license to publish the Supplemental Material files. Please contact the corresponding author directly for reuse.
