# Supplementary material for: Nitric oxide signaling through three receptors regulates virulence, biofilm formation, and phenotypic heterogeneity of Legionella pneumophila
Source: mBio. 2024 Apr 29;15(6):e00710-24. doi: 10.1128/mbio.00710-24 (PMC11237717; doi:10.1128/mbio.00710-24)
Supplement: Supplemental material. — Fig. S1-S6; Table S1. [file mbio.00710-24-s0001.pdf]

Supplementary Information

Nitric oxide signaling through three receptors regulates virulence, biofilm formation, and phenotypic heterogeneity of *Legionella pneumophila*

Sarah Michaelis[1], Tong Chen[1], Camille Schmid[1] and Hubert Hilbi[1]*

[1]*Institute of Medical Microbiology, University of Zürich, Gloriastrasse 30, 8006 Zürich, Switzerland.*

Running title: NO signaling in *Legionella*

Key words: Amoeba, *Acanthamoeba*, biofilm, cell-cell communication, flagellum, host-pathogen interaction, inter-kingdom signaling, intracellular replication, *Legionella*, macrophage, microcolony, nitric oxide, phenotypic heterogeneity, quorum sensing, timer.

Abbreviations: c-di-GMP, cyclic di-guanosine monophosphate; DKO/TKO, double/triple knockout; DPTA (dipropylenetriamine) NONOate, (Z)-1-[N-(3-aminopropyl)-N-(3-ammoniopropyl)amino]diazen-1-ium-1,2-diolate; GFP, green fluorescent protein; Hnox1, haem-nitric oxide/oxygen binding domain; Icm/Dot, intracellular multiplication/defective organelle trafficking; LAI-1, *Legionella* autoinducer-1; LCV, *Legionella*-containing vacuole; Lqs, *Legionella* quorum sensing; LvbR; *Legionella* virulence and biofilm regulator; NO, nitric oxide; NosP, NO sensing protein; SNP, sodium nitroprusside; T4SS, type IV secretion system.

***Correspondence:** E-mail hilbi@imm.uzh.ch,

Tel.: +41 (0)44 634 2650, Fax: +41 (0)44 634 4906

SUPPLEMENTARY FIGURES

Figure S1

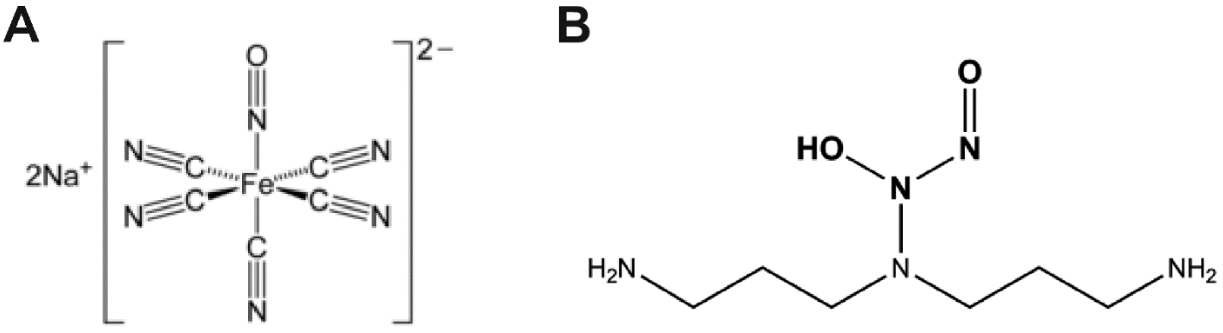

Fig. S1. Chemical structures of NO donors used in this study. The chemical structures of the NO donors (**A**) sodium nitroprusside (SNP) and (**B**) dipropylenetriamine (DPTA) NONOate. The NO groups released by DPTA NONOate are shown in bold.

Fig. S2 (overleaf)**. Effect of SNP on P*flaA*-*gfp* or P*6SRNA*-*gfp* expression in *L. pneumophila*.** (**A**) *L. pneumophila* JR32 or Δ*lqsR* harboring the P*flaA*-*gfp* (pCM009) reporter construct were grown in AYE medium for 18 h at 37°C, inoculated in AYE medium at an initial OD_{600} of 0.2 and were grown at 30°C without (black) or with 2.5 µM (green) or 5.0 µM SNP (red). GFP fluorescence and OD_{600} were measured over time using a microplate reader. Promoter activity is inferred by *gfp* expression levels, denoted as relative fluorescence units divided by Log_{10}(bacterial counts) (RFU/Log_{10}(BC)). Data shown are means and standard deviations of technical triplicates and representative of two independent experiments. *L. pneumophila* JR32 strains harboring (**B**, **C**) P*flaA*-*gfp* (pCM009) or (**D**, **E**) P*6SRNA*-*gfp* (pRH049) were grown in AYE medium without addition (black) or supplemented with 2.5 µM (green) or 5.0 µM of SNP (red). Every 2 h from 18-28 h, the bacteria were fixed with PFA and stained with DAPI. The GFP signal was determined by flow cytometry. (**B**, **D**) Percentage of GFP-positive cells after restrictive gating of the bacterial population at every timepoint. Data shown are means and standard deviations of biological triplicates (**p* < 0.05, ***p* < 0.01, ****p* < 0.001, *****p* < 0.0001; two-way ANOVA). (**C**, **E**) Representative histogram of the GFP signal of the whole bacterial population at every timepoint.

Figure S2

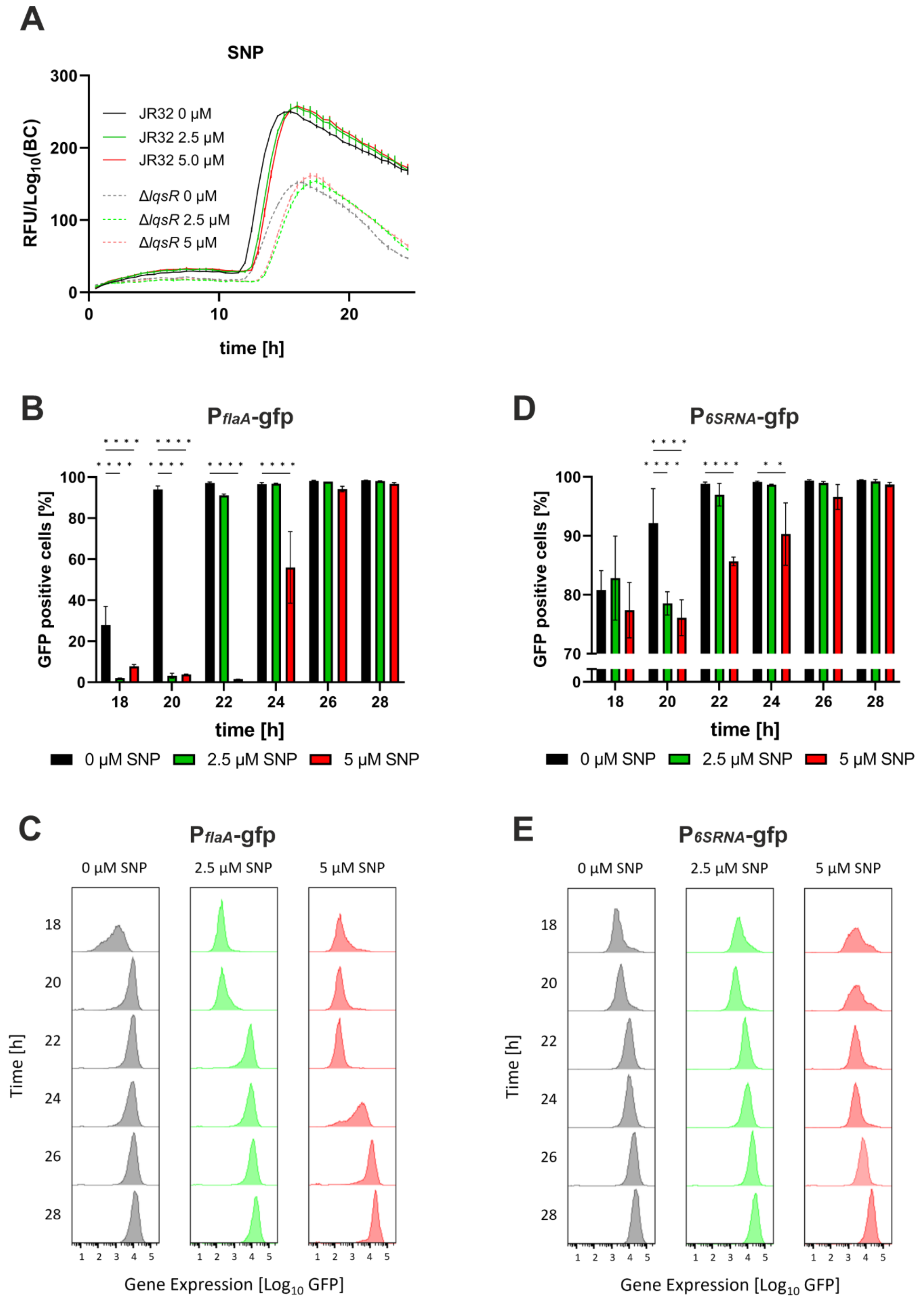
A
SNP
RFU/Log10(BC)
time [h]
JR32 0 µM
JR32 2.5 µM
JR32 5.0 µM
ΔlqsR 0 µM
ΔlqsR 2.5 µM
ΔlqsR 5 µM
B
PflaA-gfp
GFP positive cells [%]
time [h]
0 µM SNP
2.5 µM SNP
5 µM SNP
D
P6SRNA-gfp
GFP positive cells [%]
time [h]
0 µM SNP
2.5 µM SNP
5 µM SNP
C
PflaA-gfp
0 µM SNP
2.5 µM SNP
5 µM SNP
Time [h]
Gene Expression [Log10 GFP]
E
P6SRNA-gfp
0 µM SNP
2.5 µM SNP
5 µM SNP
Time [h]
Gene Expression [Log10 GFP]

Figure S3

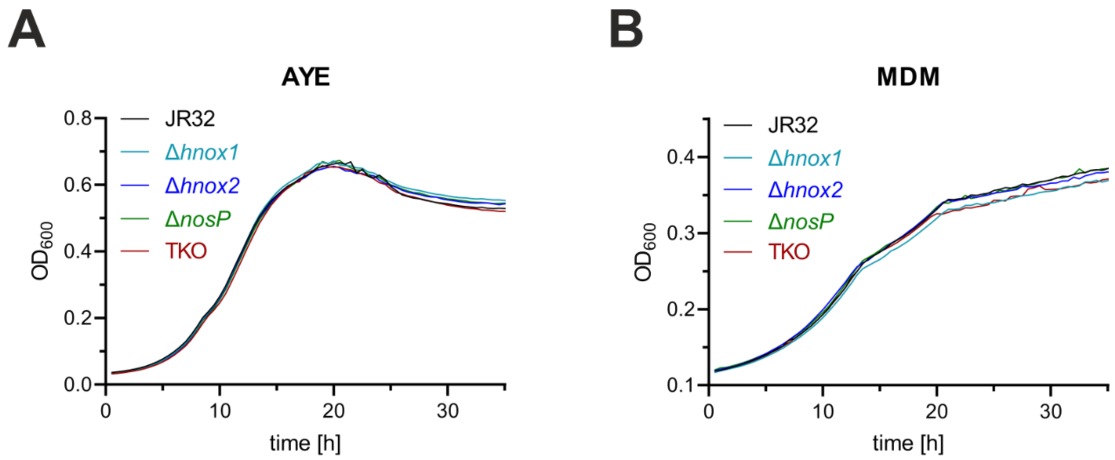

Fig. S3. Growth of *L. pneumophila* NO receptor deletion strains in medium. *L. pneumophila* JR32, Δ*hnox1*, Δ*hnox2*, Δ*nosP* or the TKO mutant strains were grown in (**A**) AYE medium or (**B**) minimal defined medium (MDM), and OD_{600} was monitored over time. Data shown are means and standard deviations of technical triplicates and representative of three independent experiments.

Figure S4

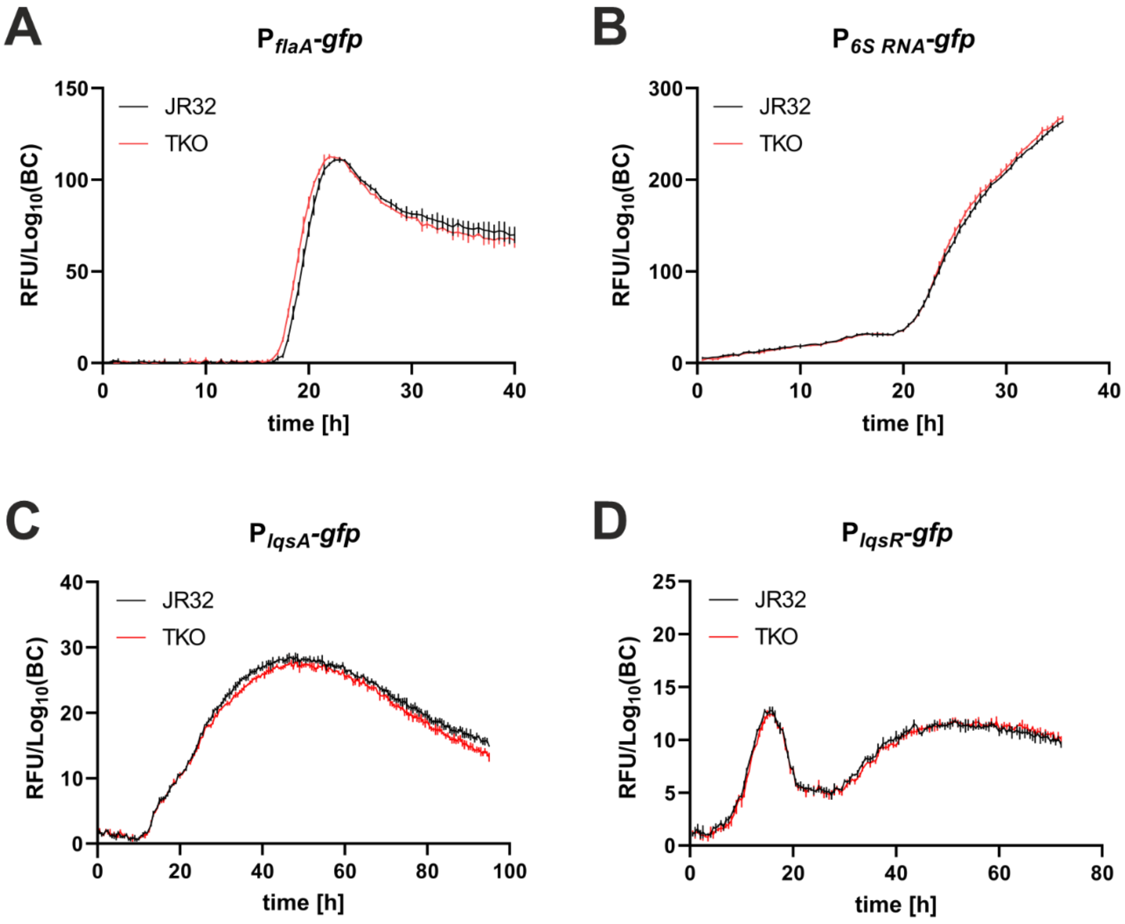

Fig. S4. **Promoter activity in *L. pneumophila* JR32 and TKO mutants.** *L. pneumophila* JR32 or TKO mutants harboring (**A**) P_{flaA}-*gfp* (pCM009), (**B**) P_{6SRNA}-*gfp* (pRH049), (**C**) P_{lqsA}-*gfp* (pRH038), or (**D**) P_{lqsR}-*gfp* (pRH037) were grown in AYE medium, and GFP fluorescence was monitored over time. Promoter activity is inferred from *gfp* expression levels, denoted as relative fluorescence units divided by log_{10}(bacterial counts) (RFU/log_{10}(BC)). Data shown are means and standard deviations of technical triplicates and representative of three independent experiments each.

Figure S5

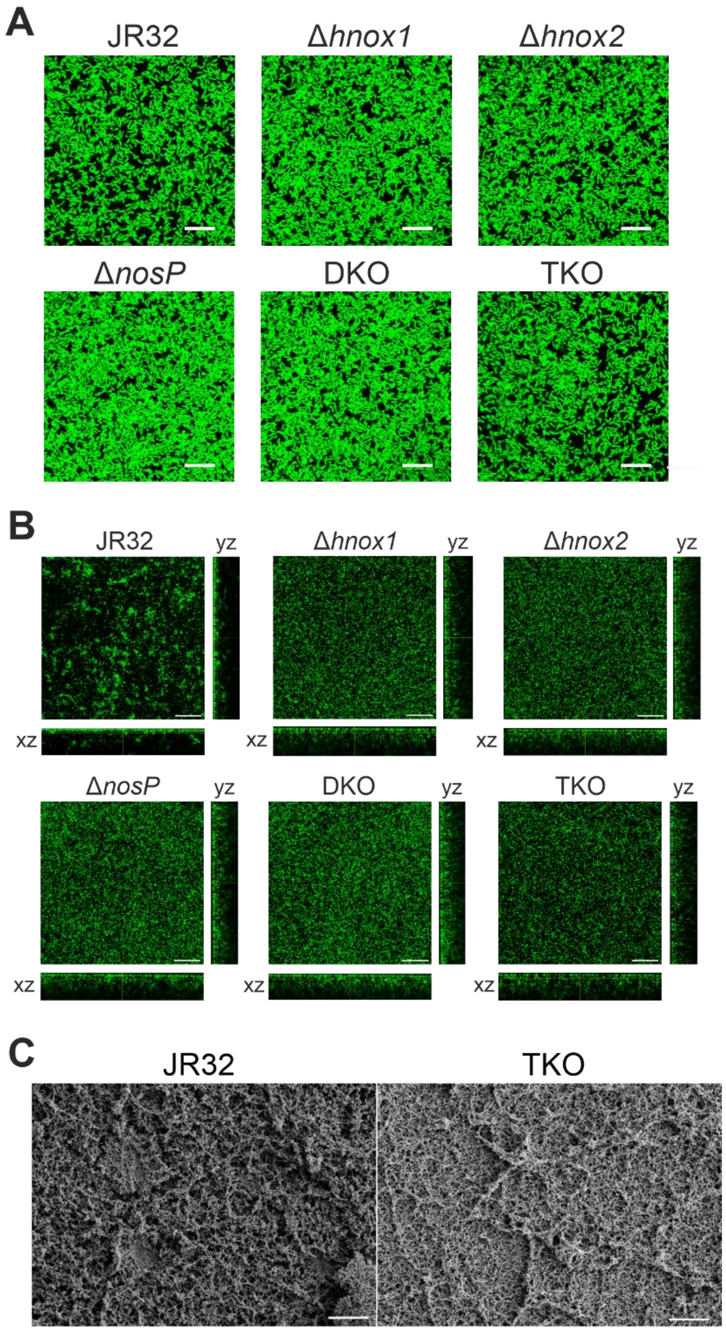

Fig. S5. Biofilm formation of *L. pneumophila* TKO mutant strain. (**A**) Exponential phase (18 h) GFP-producing *L. pneumophila* JR32, Δ*hnox1*, Δ*hnox2*, Δ*nosP* or TKO mutant strains harboring pNT28 were grown in ibiTreat microscopy dishes in AYE medium for 24 h. Confocal microscopy pictures were obtained (**A**) at the dish bottom (0 µm) to determine attachment of the strains to an abiotic surface and (**B**) at 4 µm above dish bottom (shown as cross-section of the xz and yz profile generated from z-stacks). Scale bars, 10 µm (A), 30 µm (B). The images shown are representative of at least 3 independent experiments. (**C**) Electron micrographs of *L. pneumophila* JR32 and TKO mutant biofilms. Biofilms grown for 24 h were fixed with glutaraldehyde (2.5%) in cacodylate buffer, dehydrated, dried, and coated with platinum before imaging by scanning electron microscopy. Scale bars, 60 µm.

Figure S6

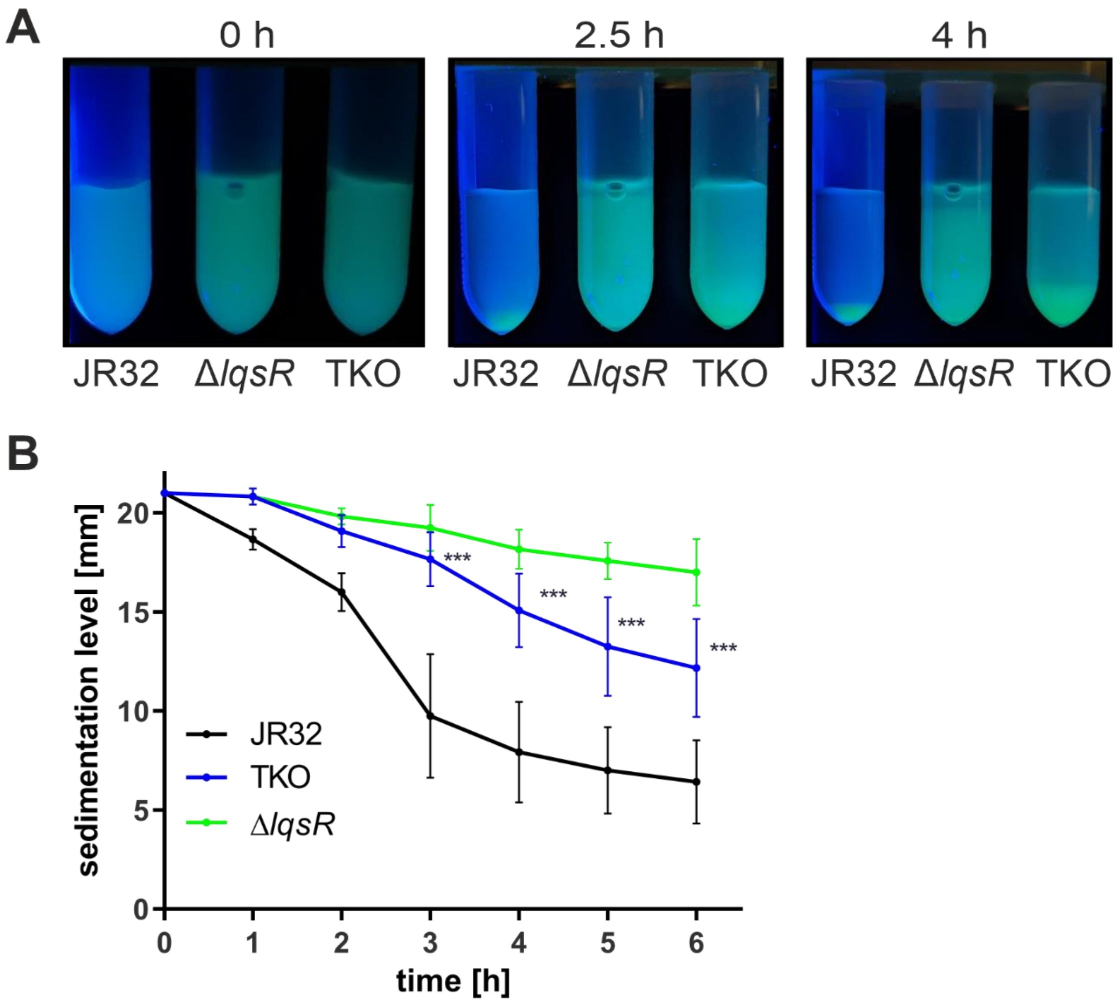

Fig. S6. Sedimentation and formation of extracellular filaments of *L. pneumophila* TKO mutant strain. (**A**) GFP-producing *L. pneumophila* JR32, Δ*lqsR* or TKO harboring pNT28 were grown for 4 d on CYE agar plates, gently suspended in AYE medium at an OD_{600} of 5.0 and let sediment at room temperature for the time indicated. Sedimentation was visualized by recording the bacterial fluorescence using a UV lamp. (**B**) Sedimentation kinetics (mm) over a period of 6 h. Data shown are means and standard deviations of six biological replicates (***, $p < 0.001$; two-way ANOVA comparing JR32 and TKO).

SUPPLEMENTARY TABLE

Table S1. Oligonucleotides used in this study.

**Oligonu-cleotide**	**Sequence 5' - 3'** [a]	**Comments**
oCS103	CAGGAAACAGAATTCGAGCTCAACAGGGGAATATCAGAAAAGTAG	Amplification of P_{hnox2}, overlap to pCM009 (fo)
oCS104	GCTCATATGTATATCTCCTTCTTAAATCTAGAGATGTTTTATATCAATTGATTAATACTATAAGTATAG	Amplification of P_{hnox2}, overlap to pCM009 (rev)
oCS105	CAGGAAACAGAATTCGAGCTCGCAACTTTAAAAAATGGCAAAATC	Amplification of P_{nosP} , overlap to pCM009 (fo)
oCS106	GCTCATATGTATATCTCCTTCTTAAATCTAGAATTGCTGTGACTTGGATTTATC	Amplification of P_{nosP} , overlap to pCM009 (rev)
oSM059	GTCGATCGCCCGG***GGATCC***AGTTTTTAGTTATTTTCCTCTTGAAG	Amplification of $P_{lpg1056}$-*lpg1056* (fo)
oSM060	CATTACGCGT***CTCGAG***GATCCTCACTCAAAGGTAATCTCCA	Amplification of $P_{lpg1056}$-*lpg1056* (rev)
oSM061	GTCGATCGCCCGG***GGATCC***GTATCTGAAAAATTATTGGGCAATTG	Amplification of $P_{lpg0279}$-*lpg0279* (fo)
oSM062	CATTACGCGT***CTCGAG***GATCCTTATGGACTCTCTAACAGGGTC	Amplification of $P_{lpg0279}$-*lpg0279* (rev)
oSM063	GTCGATCGCCCGG***GGATCC***TATATACAAAAATTAAAAGAAATGTTTACTCAAATG	Amplification of $P_{lpg2459}$-*lpg2459* (fo)
oSM064	CATTACGCGT***CTCGAG***GATCCTCACTCATCAGGATCGCC	Amplification of $P_{lpg2459}$-*lpg2459* (rev)
oSM113	GGATCCCCCGGGCTGCAGGAATTCG	pSR47S linearization (fo)
oSM114	CCACTAGTTCTAGAGCGGCCGCC	pSR47S linearization (re)
oSM124	GCTGGAGCTCCACCGCGGTGGCGGCCGCTCTAGAACTAGTGGTGTCCTTTTTATTGTTTTCCTGC	*hnox1* ± 950 bp with overlap to pSR47S (fo)
oSM125	GGTATCGATAAGCTTGATATCGAATTCCTGCAGCCCGGGGGATCCACCCATTCACCAATTGGAATAATC	*hnox1* ± 950 bp with overlap to pSR47S (re)
oSM126	CTGAAGACCATAGATGATCATTGTCGTTTGGAGATTACCTTTGAGTG	Exclusion of *hnox1* from pSM036 (fo)

oSM127	GACAATGATCATCTATGGTCTTCAGCATAAAACAA ATCTTTAAATCCC	Exclusion of *hnox1* from pSM036 (re)
oSM130	GCTGGAGCTCCACCGCGGTGGCGGCCGCTCTAGAA CTAGTGGTCTCTCACCTTGGCAGC	*nosP* ± 999 bp with overlap to pSR47S (fo)
oSM131	GGTATCGATAAGCTTGATATCGAATTCCTGCAGCC CGGGGGATCCGCATTAAGAATCACATCATGGATTG	*nosP* ± 999 bp with overlap to pSR47S (re)
oSM132	CAAGTCACAGCAATACCAAAAAGGAATTCAGTAA TAATGACAGAAATG	Exclusion of *nosP* from pSM037 (fo)
oSM133	ATTCCTTTTTGGTATTGCTGTGACTTGGATTTATCT TATGATTAATTATAG	Exclusion of *nosP* from pSM037 (re)
oSM134	GCTGGAGCTCCACCGCGGTGGCGGCCGCTCTAGAA CTAGTGGGTAATGATTTGGGGTAAGTTGATAG	*hnox2* ± 950 bp with overlap to pSR47S (fo)
oSM135	GGTATCGATAAGCTTGATATCGAATTCCTGCAGCC CGGGGGATCCAGTAATTTGGCCGTTGGC	*hnox2* ± 950 bp with overlap to pSR47S (re)
oSM136	CAATTGATATAAAACATCCTGATGAGTGATTATGA AGTATTGTTACG	Exclusion of *hnox2* from pSM038 (fo)
oSM137	CATAATCACTCATCAGGATGTTTTATATCAATTGAT TAATACTATAAGTATAGTCG	Exclusion of *hnox2* from pSM038 (re)

[a] Restriction sites are in italics and bold, regions overlapping with destination vector pCM009 or pSR47S are underlined.
